# Toward Bifunctional Chelators for Thallium-201 for Use in Nuclear Medicine

**DOI:** 10.1021/acs.bioconjchem.2c00284

**Published:** 2022-07-08

**Authors:** Alex Rigby, George Firth, Charlotte Rivas, Truc Pham, Jana Kim, Andreas Phanopoulos, Luke Wharton, Aidan Ingham, Lily Li, Michelle T Ma, Chris Orvig, Philip J. Blower, Samantha Y.A. Terry, Vincenzo Abbate

**Affiliations:** † School of Biomedical Engineering and Imaging Sciences, King’s College London, 4th Floor Lambeth Wing, St Thomas’ Hospital, London SE1 7EH, United Kingdom; ‡ Department of Chemistry, Molecular Sciences Research Hub, Imperial College London, London W12 0BZ, United Kingdom; § Medicinal Inorganic Chemistry Group, Department of Chemistry, University of British Columbia, Vancouver, BC V6T 1Z1, Canada; ∥ Life Sciences Division, TRIUMF, 4004 Wesbrook Mall, Vancouver, BC V6T 2A3, Canada; ⊥ School of Cancer & Pharmaceutical Sciences, King’s College London, Franklin-Wilkins Building, Stamford Street, London SE1 9NH, United Kingdom

## Abstract

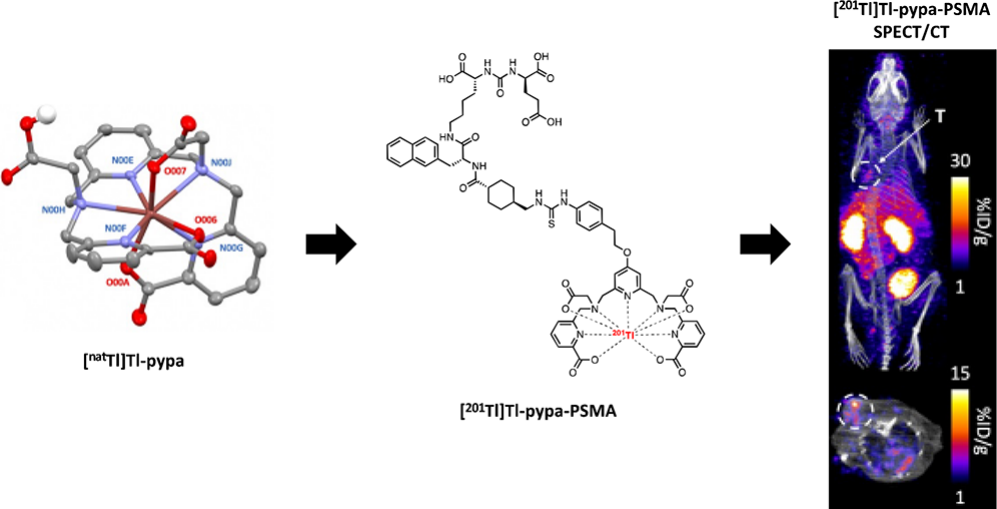

Auger electron therapy exploits the cytotoxicity of low-energy electrons emitted during radioactive decay that travel very short distances (typically <1 μm). ^201^Tl, with a half-life of 73 h, emits ∼37 Auger and other secondary electrons per decay and can be tracked *in vivo* as its gamma emissions enable SPECT imaging. Despite the useful nuclear properties of ^201^Tl, satisfactory bifunctional chelators to incorporate it into bioconjugates for molecular targeting have not been developed. H_4_pypa, H_5_decapa, H_4_neunpa-NH_2_, and H_4_noneunpa are multidentate N- and O-donor chelators that have previously been shown to have high affinity for ^111^In, ^177^Lu, and ^89^Zr. Herein, we report the synthesis and serum stability of [^nat/201^Tl]Tl^3+^ complexes with H_4_pypa, H_5_decapa, H_4_neunpa-NH_2_, and H_4_noneunpa. All ligands quickly and efficiently formed complexes with [^201^Tl]Tl^3+^ that gave simple single-peak radiochromatograms and showed greatly improved serum stability compared to DOTA and DTPA. [^nat^Tl]Tl-pypa was further characterized using nuclear magnetic resonance spectroscopy (NMR), mass spectroscopy (MS), and X-ray crystallography, showing evidence of the proton-dependent presence of a nine-coordinate complex and an eight-coordinate complex with a pendant carboxylic acid group. A prostate-specific membrane antigen (PSMA)-targeting bioconjugate of H_4_pypa was synthesized and radiolabeled. The uptake of [^201^Tl]Tl-pypa-PSMA in DU145 PSMA-positive and PSMA-negative prostate cancer cells was evaluated *in vitro* and showed evidence of bioreductive release of ^201^Tl and cellular uptake characteristic of unchelated [^201^Tl]TlCl. SPECT/CT imaging was used to probe the *in vivo* biodistribution and stability of [^201^Tl]Tl-pypa-PSMA. In healthy animals, [^201^Tl]Tl-pypa-PSMA did not show the myocardial uptake that is characteristic of unchelated ^201^Tl. In mice bearing DU145 PSMA-positive and PSMA-negative prostate cancer xenografts, the uptake of [^201^Tl]Tl-pypa-PSMA in DU145 PSMA-positive tumors was higher than that in DU145 PSMA-negative tumors but insufficient for useful tumor targeting. We conclude that H_4_pypa and related ligands represent an advance compared to conventional radiometal chelators such as DOTA and DTPA for Tl^3+^ chelation but do not resist dissociation for long periods in the biological environment due to vulnerability to reduction of Tl^3+^ and subsequent release of Tl^+^. However, this is the first report describing the incorporation of [^201^Tl]Tl^3+^ into a chelator–peptide bioconjugate and represents a significant advance in the field of ^201^Tl-based radiopharmaceuticals. The design of the next generation of chelators must include features to mitigate this susceptibility to bioreduction, which does not arise for other trivalent heavy radiometals.

## Introduction

Molecular radionuclide therapy (MRT) involves the delivery of a lethal dose of ionizing radiation emitted by a radionuclide specifically to diseased tissues or tumors. For example, α (such as ^225^Ac) and β^−^ (e.g., ^177^Lu, ^90^Y) emitting radionuclides, attached to antibodies and peptides targeting the prostate-specific membrane antigen (PSMA), have recently shown clinical promise for treating prostate cancer.^1−4^ PSMA is expressed on normal prostate cells, but its expression is greatly increased in malignant prostate tissues while remaining low in most other healthy tissues, making it a useful target for MRT.^5^ Following treatment with [^177^Lu]Lu-PSMA-617, 70% of patients experienced a decline in prostate-specific antigen (PSA) levels in the blood.^2^ A similar response was observed using [^225^Ac]Ac-PSMA-617, where patients saw a decline of ≥50% in PSA levels, which is closely associated with better overall survival.^3^

Because their typical range in tissues greatly exceeds cellular dimension, β^−^ particles are highly effective at damaging large tumors through the crossfire effect but are much less effective against single tumor cells and small cell clusters.^6,7^ In comparison, α particles and radionuclides emitting Auger electrons (AEs) have a high linear energy transfer (LET) (80–100 and 4–26 keV/μm, respectively), potentially enabling them to target and kill micrometastases and circulating tumor cells.^8,9^ α and β^−^ particles travel 40–80 μm and 0.1–10 mm, respectively, which can lead to off-target tissue toxicity to healthy tissues. This can be partially mitigated by choosing radionuclides with emissions that match the tumor size.^9^ AEs, on the other hand, travel typically <1 μm, making the likelihood of off-target effects much lower. AE-emitters thus make an exciting group of radionuclides for potentially effective MRT of micrometastases, with few side effects. This is exemplified by a recent report detailing *in vitro* and preclinical cytotoxic and antitumor effects of AE-emitting [^125^I]I-DCIBzL as a prostate cancer therapy in preclinical mouse models.^8^^161^Tb has also shown therapeutic efficacy through the emission of both beta particles and AEs. *In vivo* studies using [^161^Tb]Tb-PSMA-617 showed an improved antitumor effect compared to [^177^Lu]Lu-PSMA-617 despite the two agents having comparable pharmacokinetics.^10^ Furthermore, Vallis *et al.* have used [^111^In]In-DTPA-hEGF in Phase 1 clinical trials with 16 patients with metastatic EGFR-positive breast cancer.^11^ Radiation doses to the kidney and liver were within radiation toxicity limits, and high tumor accumulation was observed; however, for a therapeutic effect, dose escalation will be required.^11^ Michel and co-workers have highlighted the therapeutic potential of antibodies labeled with ^67^Ga, as more potency was observed when compared to ^111^In and ^125^I.^12^ Pirovano *et al.* have developed an ^123^I-labeled PARP1 inhibitor ([^123^I]I-MAPi) utilizing the Auger electron emissions as the basis of a potent radiotherapeutic for use in glioblastoma tumors.^13,14^

Thallium-201 (^201^Tl, *t*_1/2_ = 73 h) has the potential to be a highly effective therapeutic radionuclide in future MRT applications, as it emits 37 Auger and other high LET secondary electrons per decay (c.f. 25 and 12 AEs emitted by ^125^I and ^161^Tb, respectively).^9,15^ Like other AE-emitters, ^201^Tl could also facilitate a theranostics and personalized approach with accurate dosimetry as it releases gamma and X-rays, enabling single photon emission computed tomography (SPECT) imaging. Historically, ^201^Tl has been used as a SPECT myocardial perfusion imaging agent but has been largely phased out since the introduction of ^99m^Tc agents like tetrofosmin and sestamibi.

We have recently shown that nontargeted delivery of ^201^Tl (in the form of [^201^Tl]TlCl) shows short- and long-term toxicity in prostate cancer cells.^16^ A dramatic decrease in clonogenic survival was achieved at only 0.29 Bq/cell, significantly lower than for other AE-emitting radionuclides such as ^67^Ga and ^111^In.^17,18^ However, [^201^Tl]Tl^+^ has little intrinsic selectivity for tumors: it accumulates in the myocardium via the Na^+^/K^+^ ATPase pump. Thus, although it has been a very useful imaging agent for heart function, a targeted approach is required for other *in vivo* applications*.*^19^

To date, targeted delivery of ^201^Tl to cancer cells has been hindered due to the lack of suitable bifunctional chelator chemistry. Despite the high importance of ^201^Tl during the early years of nuclear medicine, thallium chelation has been poorly investigated. Previous attempts using proteins conjugated to the most common and broadly useful chelators such as DTPA or DOTA have shown complex instability.^20−22^ More recent studies carried out by our group have confirmed that Tl^3+^ complexes of EDTA, DTPA, and DOTA, despite forming Tl^3+^ complexes with very high association constants, do not possess adequate kinetic stability for MRT, highlighting the continuing need for new thallium chelators that will form kinetically stable complexes.^23^

**Figure 1:**
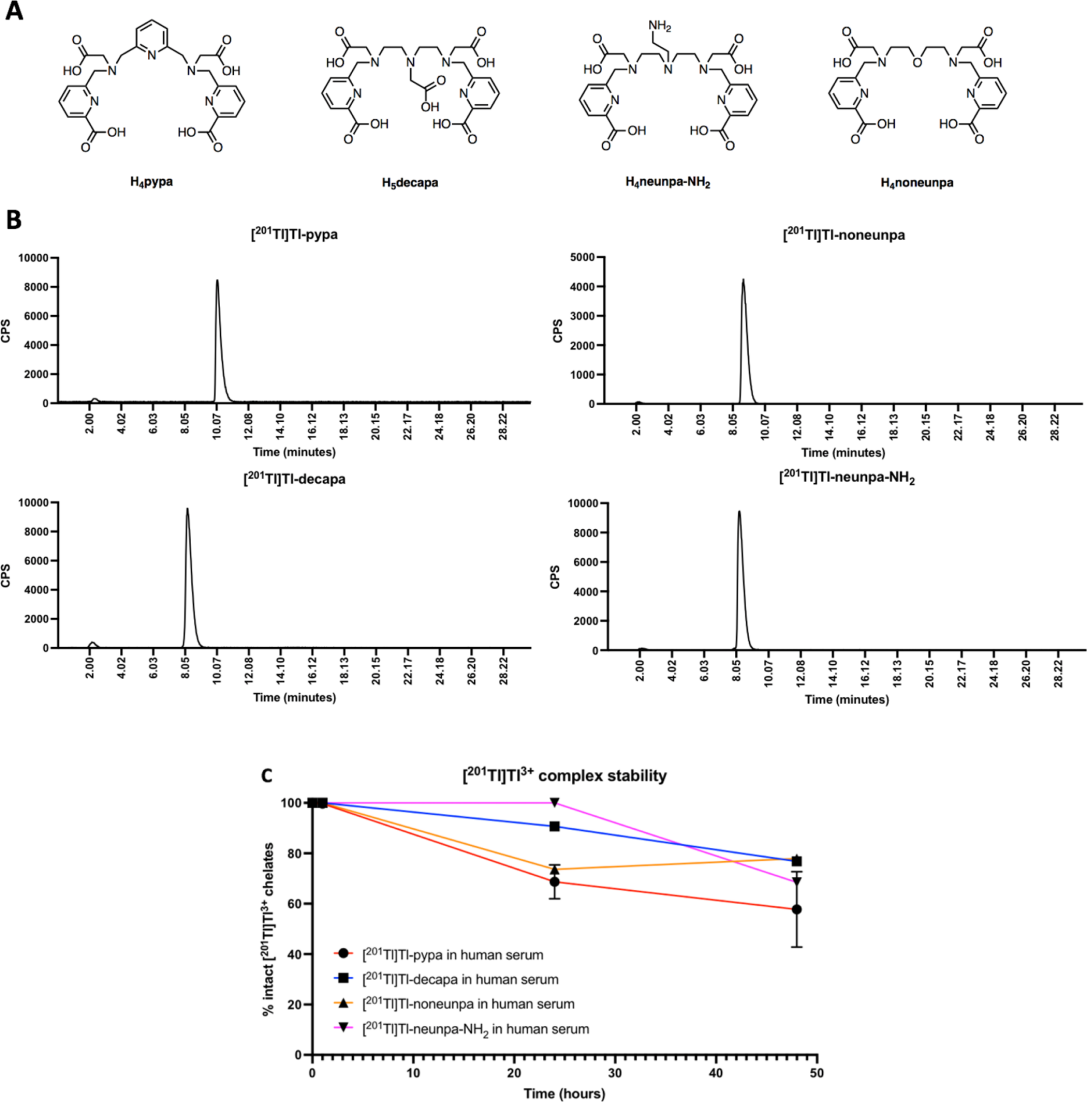
(A) Structures of H_4_pypa, H_5_decapa, H_4_neunpa-NH_2_, and H_4_noneunpa. (B) Analytical HPLC traces of [^201^Tl]Tl-pypa, [^201^Tl]Tl-decapa, [^201^Tl]Tl-neunpa-NH_2_, and [^201^Tl]Tl-noneunpa (black = counts per second) (HPLC method A). (C) Stability studies in human serum for [^201^Tl]Tl-pypa, [^201^Tl]Tl-decapa, [^201^Tl]Tl-neunpa-NH_2_, and [^201^Tl]Tl-noneunpa (*n* = 3).

Recently, Orvig and co-workers introduced a range of branched polydentate picolinic acid based chelators for evaluation as chelators for large, high-valent metal ions such as In^3+^, Lu^3+^, Sc^3+^, and Ac^3+^.^24−26^ H_4_pypa, H_5_decapa, H_4_neunpa-NH_2_, and H_4_noneunpa (Figure 1A) are chelators with a cavity size ideal for these radiometal ions, which have ionic radii (1.01–1.26 Å) similar to that of Tl^3+^ (1.12 Å).^27^ Recent studies demonstrated that H_4_pypa can also be labeled with [^44^Sc]Sc^3+^ and [^86^Y]Y^3+^ and bioconjugated to PSMA-targeting radiopharmaceuticals.^28,29^ Herein, we describe the preliminary evaluation of these ligands as chelators for [^201^Tl]Tl^3+^. As all of these chelators could be efficiently radiolabeled with [^201^Tl]Tl^3+^, we selected H_4_pypa and its previously described isothiocyanate bifunctional derivative H_4_pypa-NCS for further study. This include synthesis,*in vitro* and *in vivo* characterization of the [^201^Tl]Tl-pypa-PSMA conjugate in healthy mice and PSMA-positive and -negative tumor models in mice.^29^

## Results

### Radiolabeling Chelators

In a preliminary radiochemical screening study, we oxidized [^201^Tl]Tl^+^ to [^201^Tl]Tl^3+^ using Iodobeads and assessed radiolabeling reactions of [^201^Tl]Tl^3+^ with each of the chelators H_4_pypa, H_5_decapa, H_4_noneunpa, and H_4_neunpa-NH_2_.^23^ Each chelator (0.02 mg) was incubated with [^201^Tl]Tl^3+^ (5–10 MBq, 20–30 μL) in an aqueous solution at pH 5 at ambient temperature for 10 min followed by HPLC and RP-TLC analysis.^23^ Under the HPLC conditions employed here, [^201^Tl]Tl-pypa eluted at *t*_R_ = 10.09 min, [^201^Tl]Tl-decapa at 8.15 min, [^201^Tl]Tl-noneunpa at 8.44 min, and [^201^Tl]Tl-neunpa-NH_2_ at 8.17 min, whereas unchelated [^201^Tl]TlCl_3_ eluted earlier at 2.03 min. Radiolabeling was rapid in all cases; radiochromatograms (Figure 1B) show radiochemical yields of >97% after only 10 min of incubation at room temperature (RT). Each chelator was also evaluated with [^201^Tl]Tl^+^ (i.e., without prior treatment with Iodobeads); no complexation reaction was observed by HPLC in these experiments (Figure S3).

### *In Vitro* Stability

Each [^201^Tl]Tl-labeled complex was left standing in an ammonium acetate solution (1 M, pH 5) for 48 h (Figure S4), and each showed no degradation. However, all complexes showed modest stability when incubated in human serum at 37 °C (Figure 1C). After 24 h in human serum, 68.7 ± 6.5% of the [^201^Tl]Tl-pypa complex was intact, decreasing to 57.7 ± 15.1% after 48 h. [^201^Tl]Tl-decapa, [^201^Tl]Tl-neunpa, and [^201^Tl]Tl-noneunpa showed very similar complex stability to [^201^Tl]Tl-pypa in human serum after 48 h. In addition to serum, the stability of all four [^201^Tl]Tl-labeled complexes was evaluated in the presence of excess apotransferrin. All showed resistance to transmetalation over 24 h (Figures S5–S7).

All four evaluated chelators could be efficiently labeled with [^201^Tl]Tl^3+^, but all [^201^Tl]Tl-labeled complexes were susceptible to demetallation in serum (55–80% intact after 48 h incubation). We elected to further assess bioconjugates of H_4_pypa for [^201^Tl]Tl^3+^ receptor-targeted delivery to prostate cancer cells. A bifunctional H_4_pypa derivative, alongside bioconjugates of H_4_pypa with a monoclonal antibody and an alternative PSMA-targeted peptidic motif, has been previously described.^28−30^ We therefore based the selection of the H_4_pypa ligand for further evaluation on the ease of H_4_pypa incorporation into bioconjugates.

### Synthesis and Characterization of [Tl(Hpypa)]

**Figure 2:**
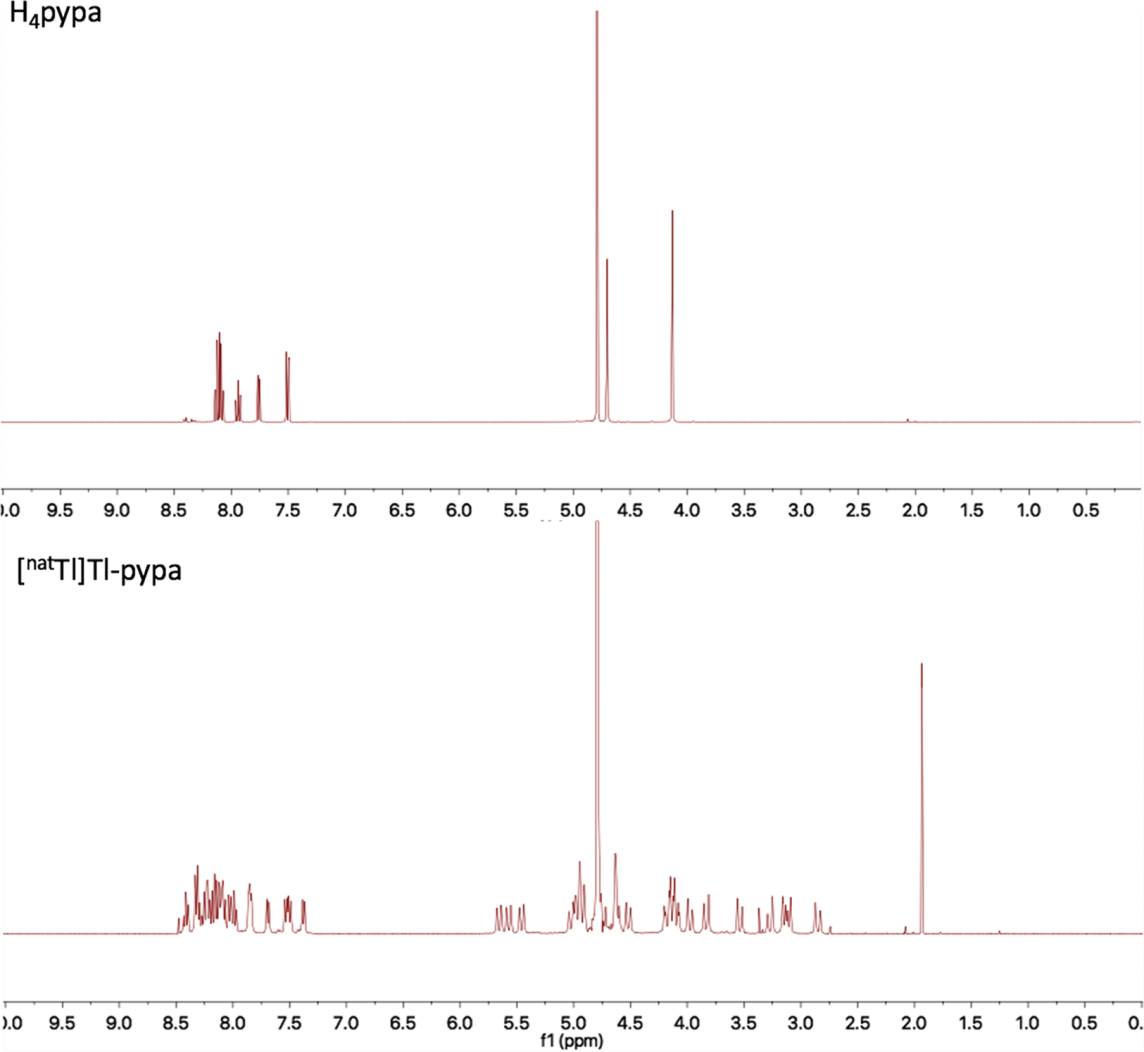
^1^H NMR spectra (D_2_O) of H_4_pypa (top) and [^nat^Tl]Tl-pypa (bottom).

The chelator H_4_pypa^30^ was reacted with thallium trichloride hydrate ([^nat^Tl]TlCl_3_(H_2_O)_4_) in an ammonium acetate solution (pH = 5) at RT for 15 min to yield [Tl(Hpypa)] as a white solid. Following purification, the complex was characterized using nuclear magnetic resonance (NMR) (Figure 2 and Figures S10–S27) and high-resolution electrospray ionization mass spectrometry (HR-ESI-MS) (Figures S28–S33). HRMS data show the formation of a 1:1 complex of H_4_pypa with Tl^3+^. Due to the poor solubility of [Tl(Hpypa)] in D_2_O, a small amount of Na_2_CO_3_ (in D_2_O) was added, adjusting the pH to 8–9. This greatly increased solubility, presumably by the formation of [Tl(pypa)]^−^, enabling NMR (^1^H, ^13^C, and 2D NMR) spectroscopic studies to be carried out. Under more acidic conditions, the [Tl(Hpypa)] complex was insufficiently soluble to obtain NMR spectra. Previous reports in the literature show that when complexed with In^3+^, Lu^3+^, and La^3+^ ions, H_4_pypa forms rigid complexes giving rise to sharp ^1^H NMR peaks suggesting little fluxionality. However, the ^1^H and COSY NMR data for [Tl(pypa)]^−^ suggest that there are at least two species in the solution (Figure 2). In complexes of pypa, methylene protons are diastereotopic, with coupling between geminal, diastereotopic methylene protons. In the ^1^H COSY spectrum (Figure S25) of the pypa complex of Tl^3+^, 12 cross peaks between methylene protons are observed, indicating that at least two chemically distinct [^nat^Tl]Tl-pypa complexes are present in the solution that do not interconvert rapidly within the NMR time scale.

**Figure 3:**
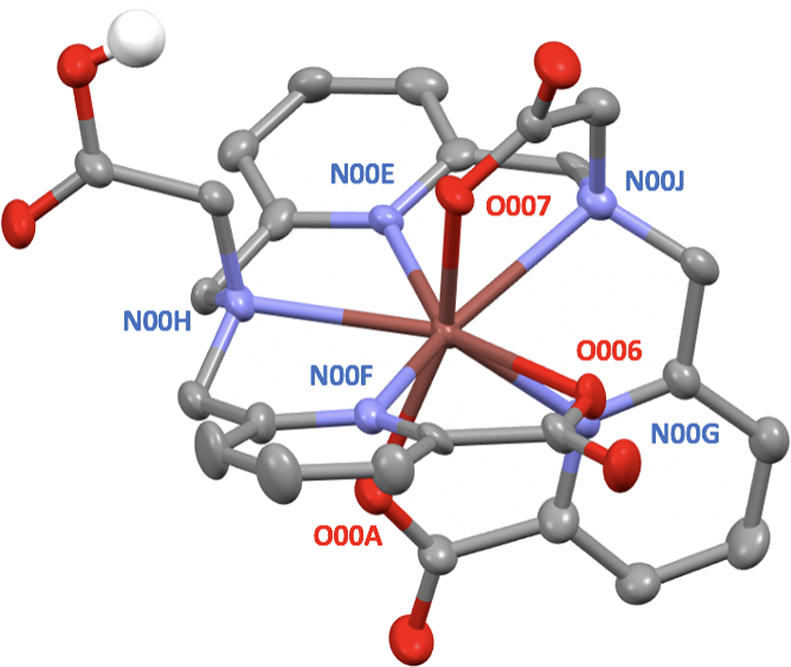
Crystal structure of [Tl(Hpypa)] (50% probability ellipsoids).

X-ray quality single crystals of [Tl(Hpypa)] were obtained by the slow evaporation of equimolar mixtures of TlCl_3_ and H_4_pypa solutions in water with the pH adjusted to 2 by the addition of HCl (0.1 M).^30^ The crystal structure of [Tl(Hpypa)] is shown in Figure 3, and selected bond lengths can be found in Table 1. Full crystallographic information can be found in Figure S34. The complex has an octacoordinated Tl^3+^ in a distorted square antiprismatic geometry, and when grown from a solution at pH 2, one of the carboxylic acid groups is protonated and does not coordinate to Tl^3+^. The Tl^3+^ ion is coordinated by eight (N_5_O_3_) of the nine potential donor atoms of the ligand. The Tl–O bond lengths are between 2.258(6) and 2.496(5) Å, and Tl–N bond lengths are between 2.311(7) and 2.525(7) Å. These are comparable to bond lengths previously reported for Tl^3+^ complexes.^31−33^

**Table 1:** Selected Bond Lengths and Angles in [Tl(Hpypa)]

bond lengths			bond angles			
atom	atom	length (Å)	atom	atom	atom	angle (°)
Tl	O006	2.496(5)	O006	Tl	N00H	136.2(2)
Tl	O007	2.258(6)	O006	Tl	N00J	82.2(2)
Tl	O00A	2.370(6)	O007	Tl	O00A	156.0(2)
Tl	N00E	2.358(6)	O007	Tl	N00E	96.5(2)
Tl	N00F	2.348(7)	N00E	Tl	O006	67.6(2)
Tl	N00G	2.311(7)	N00F	Tl	N00J	141.6(2)
Tl	N00H	2.530(6)	N00G	Tl	N00F	120.5(2)
Tl	N00J	2.525(7)	N00J	Tl	N00H	129.8(2)

A low symmetry is observed due to the uncoordinated carboxyl group. Numerous attempts were made to grow an X-ray quality crystal at neutral pH or with an alternative counter ion, for example, tetrabutylammonium, but were not fruitful. Under more basic conditions, it is possible that both carboxylate groups coordinate the metal ion, allowing for a higher degree of symmetry.

### Synthesis of H_4_pypa-PSMA

As a basis for bioconjugate synthesis, an isothiocyanate derivative of H_4_pypa, H_4_pypa-NCS, was synthesized using the method previously described by Li *et al*.^28,30^ To deliver ^201^Tl to PSMA-expressing cells, H_4_pypa-NCS must be coupled to the PSMA targeting vector via a linker molecule. Structure–activity relationships (SARs) of several PSMA targeting variants have demonstrated the significant role that linker design can have on the pharmacokinetic profile of a tracer.^34^ The linker used here, incorporating a naphthyl group, was chosen due to the desirable characteristics of PSMA-617 *in vivo*,^34^ including the high affinity for PSMA (assisted by the lipophilic linker binding to the hydrophobic PSMA pocket) and fast renal clearance shown by derivative PSMA-617.^34^

**Scheme 1:**
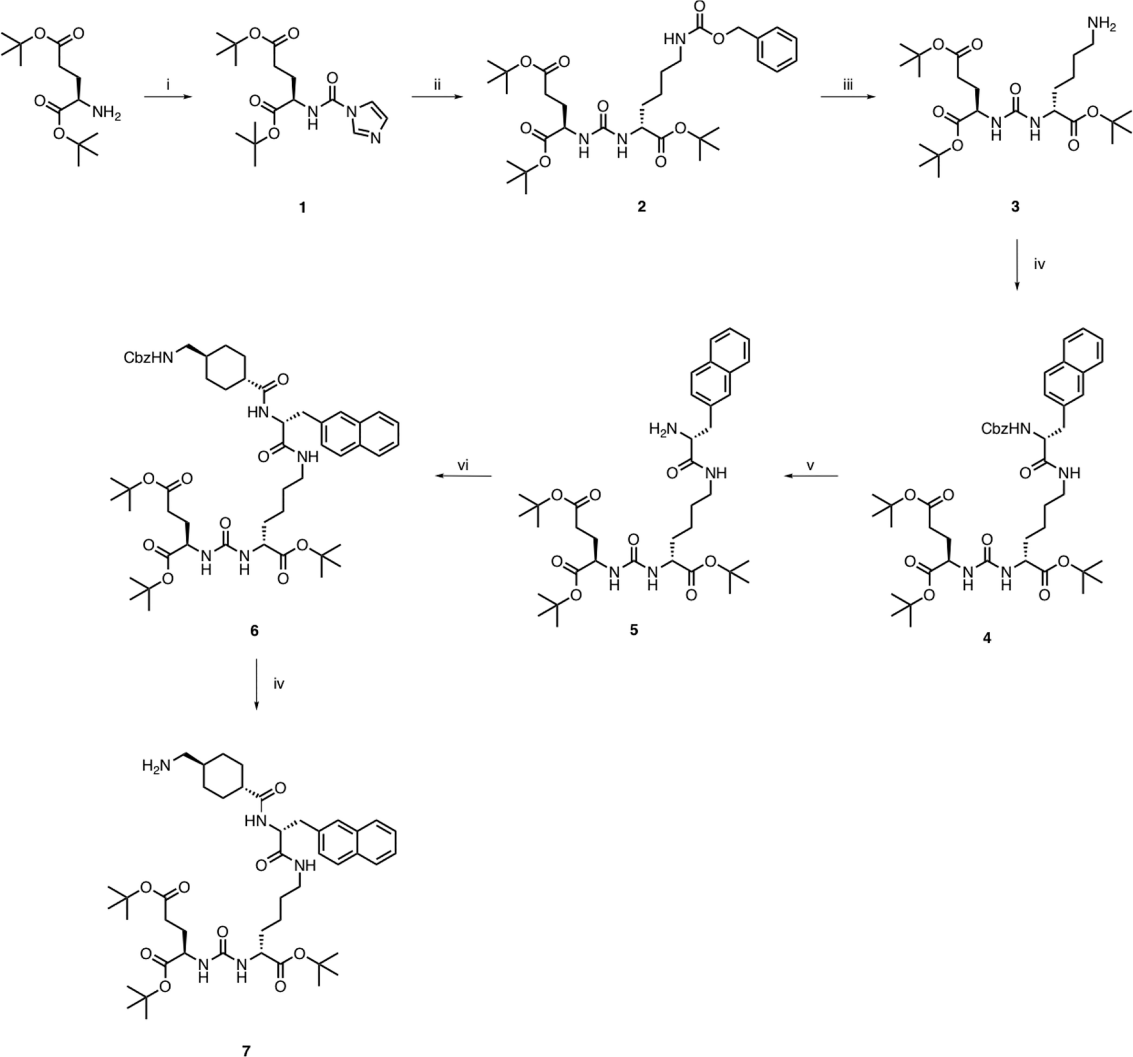
Reagents and Conditions for the Synthesis of Compounds **1**–**7** (i) CDI, MeCN/DMF (4:1), RT, 24 h, 51%. (ii) H-Lys(cbz)-OtBu, DIPEA, DMF, RT, overnight, 83%. (iii) Pd/C, MeOH, RT, overnight, 92%. (iv) Cbz-3-(2-naphthyl)-d-alanine, HATU, DIPEA, DMF, RT, overnight, 55%. (v) Pd/C, MeOH, RT, overnight, 89%. (vi) cbz-*trans*-4-(aminomethyl)cyclohexanecarboxylic acid, HATU, DIPEA, DMF, RT, overnight, 63%. (vii) Pd/C, MeOH, RT, overnight, 91%.

To prepare the PSMA peptide analogue for coupling to H_4_pypa-NCS, we adapted a previously reported method, as shown in Scheme 1.^35^ In brief, l-glutamic acid di-*tert*-butyl ester was reacted with carbonyldiimidazole (CDI), forming the activated glutamic acid **1**. This was then reacted with the cbz-protected l-lysine *tert*-butyl ester to yield the urea **2**. The cbz group was then removed via catalytic hydrogenation, generating the urea derivative **3**. Cbz-3-(2-naphthyl)-d-alanine was added via HATU mediated amide coupling in DMF to furnish compound **4** followed by a hydrogenation reaction to remove the cbz group (**5**). The coupling and cbz deprotection procedures were repeated with cbz-*trans*-4-(aminomethyl)cyclohexanecarboxylic acid to generate **6** and **7**, respectively.

**Scheme 2:**
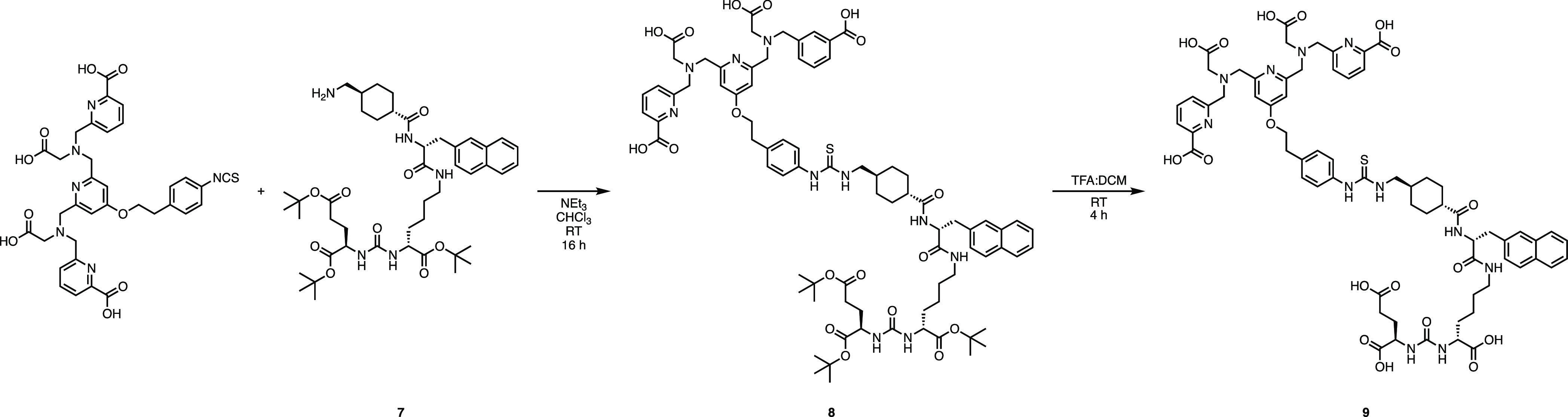
Reagents and Conditions for the Synthesis of Compounds **8** and **9** (i) CHCl_3_, NEt_3_, RT, overnight, 56%. (ii) TFA/DCM, overnight, 75%.

The reaction of a basic solution of H_4_pypa-NCS in chloroform with **7** at ambient temperature led to the formation of conjugate **8** (Scheme 2). The *tert*-butyl groups of **8** were cleaved using trifluoracetic acid in DCM (1:1) to generate H_4_pypa-PSMA (**9**), which was purified using reversed-phase HPLC. HR-MS confirmed the formation of the final product **9**.

**Figure 4:**
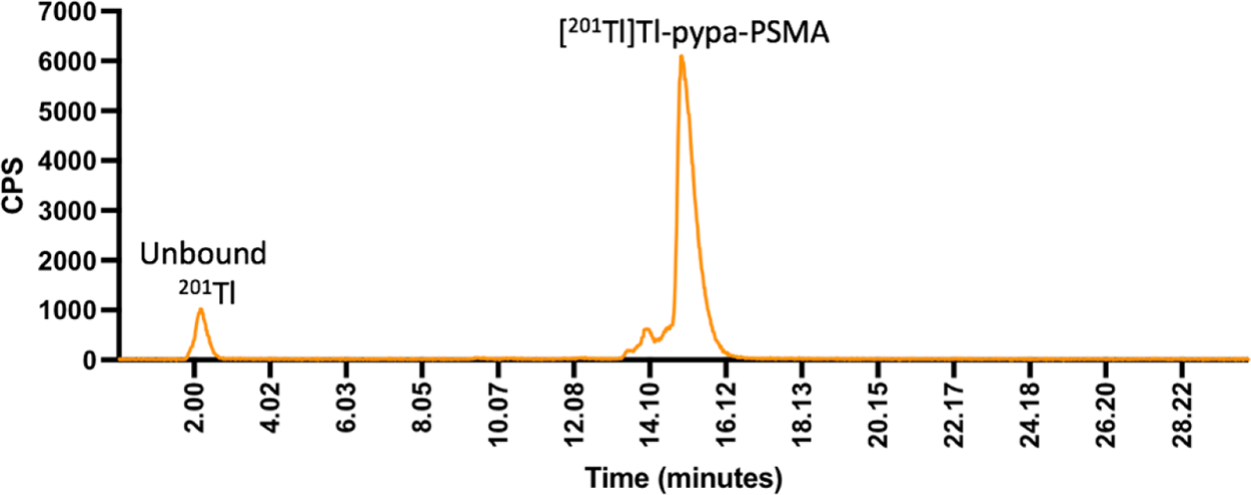
Analytical radio-HPLC trace of [^201^Tl]Tl-pypa-PSMA using the HPLC method A (orange = counts per second) (HPLC method A).

The method previously described for the radiolabeling of H_4_pypa with ^201^Tl, incorporating prior oxidation of [^201^Tl]Tl^+^ to [^201^Tl]Tl^3+^, was used to radiolabel **9** in good radiochemical yields (95 ± 3%). HPLC analysis indicated that [^201^Tl]Tl-pypa-PSMA eluted at 15.9 min (10.7–24.5 MBq, 20 mmol) (Figure 4). A HPLC UV trace of the unlabeled H_4_pypa-PSMA is included in Figure S8.

**Figure 5:**
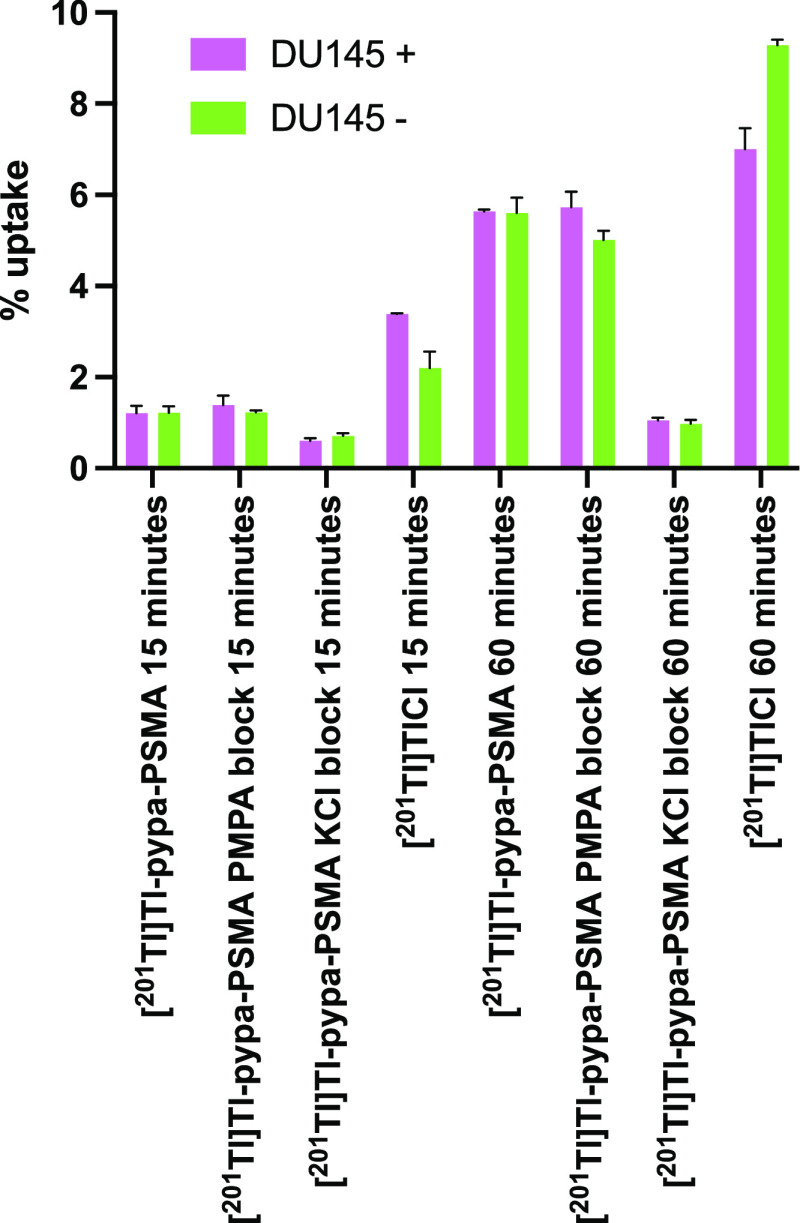
Cell uptake experiments in DU145 PSMA-positive and -negative cell lines with [^201^Tl]-pypa-PSMA or [^201^Tl]TlCl after 15 or 60 min of incubation at 37 °C.

[^201^Tl]Tl-pypa-PSMA uptake was then evaluated in DU145 PSMA-positive and PSMA-negative cells after 15 and 60 min of incubation (Figure 5). The amount of cell-associated [^201^Tl]Tl was similar for PSMA-positive and PSMA-negative cells lines, indicating that [^201^Tl]Tl accumulation is not specific to PSMA expression. Additionally, and consistent with this, co-incubation with an excess of the PSMA inhibitor PMPA (2-phosphonomethyl pentanedioic acid) did not meaningfully reduce [^201^Tl]Tl accumulation in either cell line.

The uptake of [^201^Tl]TlCl was also measured under the same conditions: the amount of ^201^Tl associated with cells was in fact higher for cells incubated with [^201^Tl]TlCl compared to cells incubated with [^201^Tl]Tl-pypa-PSMA. Lastly, co-incubation with an excess of KCl reduced the uptake of [^201^Tl]Tl-pypa-PSMA in both PSMA-positive and PSMA-negative cells lines.

Cumulatively, the data suggest that in the presence of cells, [^201^Tl]Tl-pypa-PSMA releases ^201^Tl and that this dissociation is potentially mediated by the reduction of [^201^Tl]Tl^3+^ to [^201^Tl]Tl^+^ by endogenous reductants. Released [^201^Tl]Tl^+^ then behaves as a K^+^ mimic and is taken up by both PSMA-positive and PSMA-negative cells, with accumulation (via potassium channels, including the Na^+^/K^+^-ATPase pump) inhibited by co-incubation with excess K^+^.

### *In Vivo* Biodistribution in Healthy Animals

**Figure 6:**
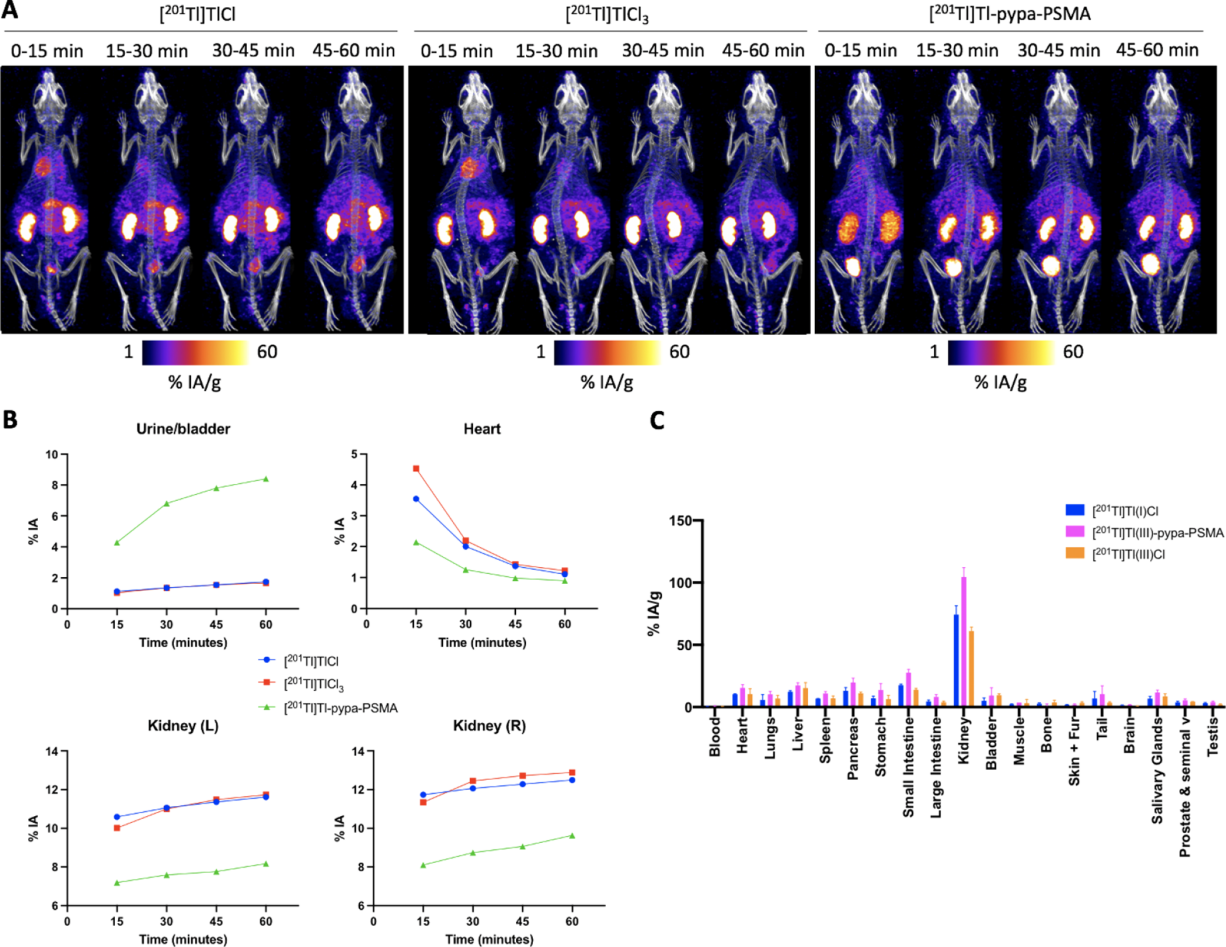
(A) *In vivo* images of [^201^Tl]TlCl, [^201^Tl]TlCl_3_, and [^201^Tl]Tl-pypa-PSMA at 15, 30, 45, and 60 min in healthy animals (*n* = 3 per group). (B) Regions of interest (ROIs) drawn from the SPECT images around organs of interest (bladder, heart, and kidneys) for [^201^Tl]TlCl, [^201^Tl]TlCl_3_, and [^201^Tl]Tl-pypa-PSMA at 15, 30, 45, and 60 min (*n* = 1 per radiotracer). (C) The *ex vivo* biodistribution of [^201^Tl]TlCl, [^201^Tl]TlCl_3_, and [^201^Tl]Tl-pypa-PSMA in healthy SCID beige mice culled at 1 h post injection (*n* = 3 per radiotracer).

To compare the biodistribution of [^201^Tl]Tl-pypa-PSMA, [^201^Tl]TlCl, and [^201^Tl]TlCl_3_, all three tracers were administered intravenously via the tail vein to healthy male SCID/beige mice. SPECT/CT images were acquired at 15 min intervals up to 1 h after injection (Figure 6A). Mice were then culled, and organs were collected for *ex vivo* biodistribution (Figure 6C).

SPECT/CT images showed that compared to [^201^Tl]Tl-pypa-PSMA, ^201^Tl administered as either Tl^+^ or Tl^3+^ has an initially high heart uptake at 15 min (4.5% and 3.6% IA (percentage injected activity), respectively) followed by washout, a high degree of retention in the kidneys (10.0–12.9% IA), and relatively low excretion via the urine/bladder (<1.7% IA at all time points) (Figure 6A). In contrast, [^201^Tl]Tl-pypa-PSMA showed a lower myocardial accumulation at 15 min (2.1% IA) and significant [^201^Tl]Tl activity associated with the urine/bladder (8.4% at 60 min).

*Ex vivo* biodistribution data showed that blood values were low for [^201^Tl]TlCl, [^201^Tl]TlCl_3_, and [^201^Tl]Tl-pypa-PSMA with only 0.24, 0.18, and 0.19% activity, respectively, present in blood at 1 h post injection (p.i.) (Figure 6C). [^201^Tl]TlCl and [^201^Tl]TlCl_3_ have a high heart uptake of 10.3 ± 0.1% injected activity per gram (IA/g) and 15.4 ± 2.6% IA/g at 1 h p.i., respectively, while [^201^Tl]Tl-pypa-PSMA showed a lower uptake (8.0 ± 0.4% IA/g) (Figure 6C), consistent with SPECT imaging analysis. All three ^201^Tl compounds were predominantly cleared via the kidneys, with [^201^Tl]TlCl having 74.4 ± 6.3% IA/g, [^201^Tl]TlCl_3_ having 104.5 ± 6.9% IA/g, and [^201^Tl]Tl-pypa-PSMA having 61.0 ± 3.0% IA/g accumulating in kidneys at 1 h p.i. Clearance through the liver was much lower for all three groups, with [^201^Tl]TlCl having 12.3 ± 0.6% IA/g, [^201^Tl]TlCl_3_ having 17.5 ± 2.0% IA/g, and [^201^Tl]Tl-pypa-PSMA having 15.3 ± 4.2% IA/g accumulating in the liver by 1 h p.i.

### [^201^Tl]Tl-pypa-PSMA in a Prostate Cancer Animal Model

The biodistribution of [^201^Tl]Tl-pypa-PSMA was studied in SCID/beige mice bearing either (i) DU145 PSMA-expressing tumors (PSMA-positive) or (ii) DU145 tumors that do not express the PSMA receptor (PSMA-negative) to determine if [^201^Tl]Tl-pypa-PSMA accumulated in prostate cancer tissues via PSMA receptor binding. This model has previously been used to show the PSMA-specific uptake of tracers.^36^ Each group of mice was administered [^201^Tl]Tl-pypa-PSMA (10.7–24.5 MBq, 20 nmol) prior to SPECT/CT scanning for 2 h. At the conclusion of the SPECT/CT scan, each mouse was culled, and organs were dissected, weighed, and counted for radioactivity to obtain quantitative data on radiotracer biodistribution.

**Figure 7:**
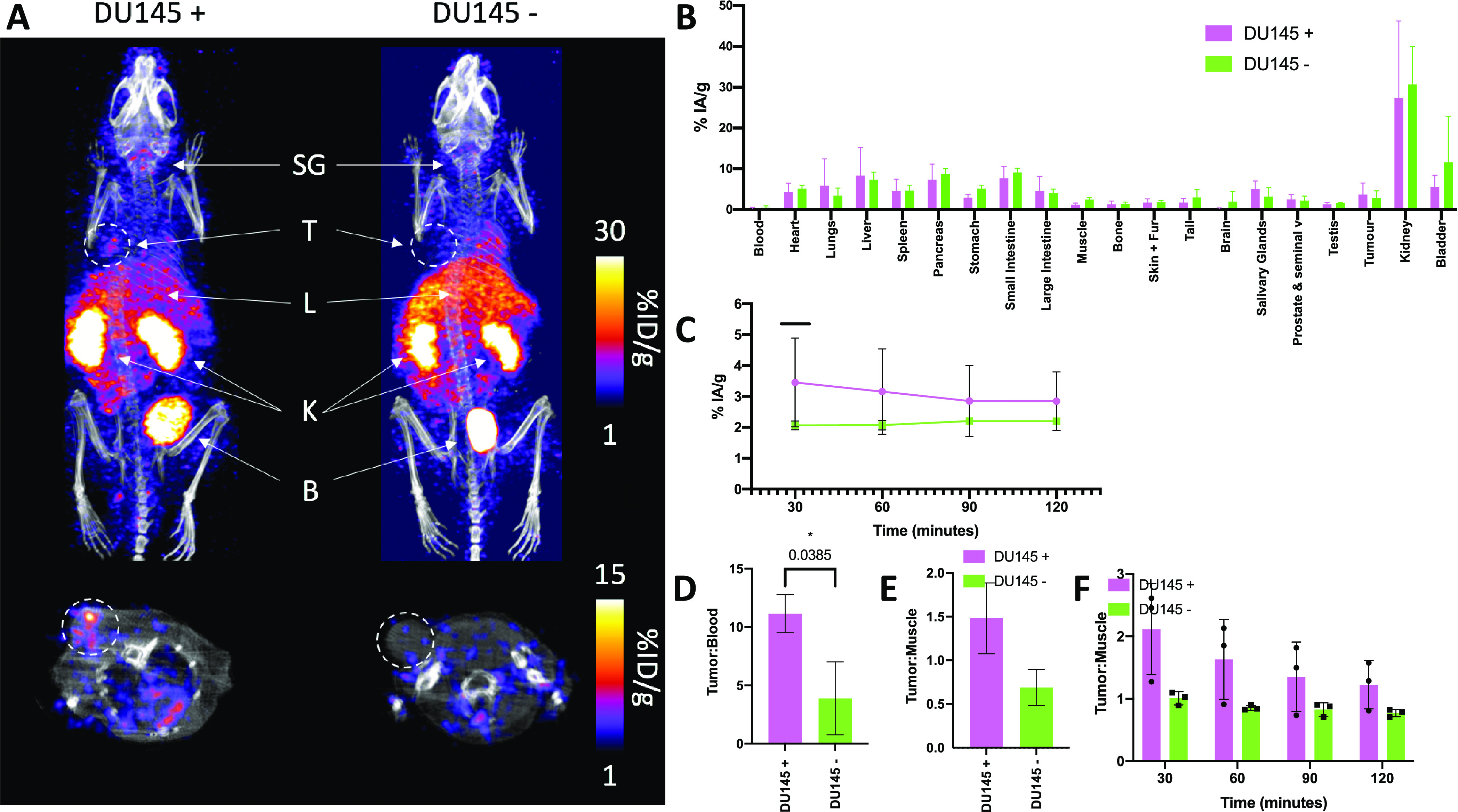
(A) *In vivo* SPECT image (0–30 min) of [^201^Tl]Tl-pypa-PSMA in mice bearing DU145 positive and negative tumors at 0–30 min. SG = salivary glands, T = tumor, L = liver, K = kidneys, and B = bladder. (B) *Ex vivo* biodistribution of [^201^Tl]Tl-pypa-PSMA in mice bearing DU145 positive and negative tumors 2 h p.i. (*n* = 3 per group). (C) Uptake in DU145 PSMA-positive and PSMA-negative tumors using regions of interest drawn from the SPECT images at 30, 60, 90, and 120 min. Tumor to blood (D) and muscle (E) ratios were calculated using biodistribution data (2 h p.i.). Tumor to blood ratios were taken from ROIs drawn on the SPECT images at various time points (F).

SPECT imaging analysis indicated that radioactivity concentration in DU145 PSMA-positive tumors was consistently higher than in DU145 PSMA-negative tumors and, at early time points only, this difference was statistically significant. At 30 min, the ^201^Tl radioactivity concentration in PSMA-positive DU145 tumors measured 3.5 ± 1.4% IA/g (*p* = 0.0219) and decreased to 2.9 ± 0.9% IA/g at 2 h p.i. (Figure 7C). For PSMA-negative DU145 tumors, ^201^Tl radioactivity concentration at 30 min was 2.1 ± 0.2% IA/g and remained steady until 2 h p.i. Biodistribution data 2 h p.i. corroborated SPECT imaging analysis: ^201^Tl concentration at 2 h p.i. in DU145 PSMA-positive tumors measured 3.7 ± 2.8% IA/g, and in the PSMA-negative tumors, this ^201^Tl radioactivity concentration measured 2.9 ± 1.5% IA/g (Figure 7B). Imaging and *ex vivo* biodistribution data further evidenced that [^201^Tl]Tl-pypa-PSMA is cleared from the blood mainly via a renal pathway, with high levels of radioactivity observed in the kidneys and bladder/urine evident in both imaging and *ex vivo* biodistribution data.

*Ex vivo* biodistribution data also indicated that the tumor/blood ratio for PSMA-positive tumors (11.1 ± 1.4) was significantly higher than that for PSMA-negative tumors (3.9 ± 3.0) at 2 h p.i. (*p* = 0.0385). The tumor/muscle ratio was similarly higher in mice bearing PSMA-positive tumors (ratio of 1.5 ± 0.4) than in mice bearing PSMA-negative tumors (ratio of 0.7 ± 0.2) (Figure 7E). SPECT image analysis was also used to determine tumor/muscle ratios for [^201^Tl]Tl-pypa-PSMA. The tumor/muscle ratio for animals bearing PSMA-negative tumors was approximately 1 from 30 min to 2 h p.i. However, the tumor/muscle ratio for animals bearing PSMA-positive tumors measured 2.1 ± 0.7 at 30 min and decreased to 1.2 ± 0.4 at 2 h p.i.

## Discussion

The premise of this work is that to explore the potential of ^201^Tl as a therapeutic radionuclide, we need better chelators for thallium, capable of both convenient radiolabeling under mild conditions and resistance to dissociation or transchelation in the biological environment. Chelation of Tl^+^ is likely to be challenging due to the similarity of its aqueous chemical properties to those of group 1 alkali metals.^37^ Therefore, in this study, we chose to focus on Tl^3+^.

Established general-purpose chelators widely used for a range of radiometals in nuclear medicine, such as DOTA and DTPA, are excellent chelators for In^3+^ (the closest periodic analogue of Tl^3+^) and indeed form well-defined complexes with Tl^3+^ with high affinity. Nevertheless, the DOTA and DTPA complexes of Tl^3+^ quickly decompose in serum and cannot be used in Tl^3+^ radiopharmaceuticals.^21^ No binding constants of either Tl^+^ or Tl^3+^ to endogenous serum proteins have been reported in the literature.^38^ However, Li *et al.* have estimated the binding of Tl^3+^ to transferrin to have an association constant of 10^22^ based on the linear relationship that they have observed between the first hydrolysis constant of the other trivalent group 13 metal ions and their transferrin binding constant.^39^ An alternative route to dissociation of Tl^3+^ complexes, not available to their In^3+^ analogues, is reduction of Tl^3+^ to Tl^+^ by reducing agents present in biological media. Because of this unique vulnerability to reduction of Tl^3+^, the analogy to In^3+^ and other trivalent heavy metals such as bismuth and lanthanides offers only limited guidance in the design of thallium chelators.

Nevertheless, as a starting point for the evaluation of chelators for Tl^3+^, we chose to evaluate a range of polydentate acyclic chelators containing amine, pyridine, and carboxylate donors (Figure 1) that have shown great promise with In^3+^ and other trivalent metal ions. Initial evaluation of the radiolabeling of these ligands with [^201^Tl]Tl^3+^, after oxidation of [^201^Tl]Tl^+^ to [^201^Tl]Tl^3+^ with Iodobeads, showed that all of them were able to chelate [^201^Tl]Tl^3+^ quickly and efficiently under mild conditions and in this respect represent an improvement on DOTA, which required longer reaction times (60 min at room temperature).^23,40^ The radiochromatograms of the labeling mixtures each showed single peaks, suggesting the absence of major isomerism or that isomers were rapidly interconvertible (although, at least in the case of the pypa complex, this interpretation is not consistent with ^1^H NMR discussed below). On this basis, all four complexes warranted the evaluation of stability in biological media.

The complexes showed no measurable dissociation when incubated in an ammonium acetate buffer or in the presence of transferrin but showed slow decomposition over hours to days in human serum. Although this rate of dissociation is suboptimal, it does not necessarily preclude the use of these chelators in ^201^Tl radiopharmaceuticals, and it is significantly better than that reported for EDTA, DTPA, and DOTA:^23,40^ [^201^Tl]Tl-DTPA decomposed quickly in human serum (<10% intact after 1 h), and only 42.7 ± 20.8% of [^201^Tl]Tl-DOTA remained intact after 24 h.^23^

The stabilities of [^201^Tl]Tl-pypa, [^201^Tl]Tl-decapa, [^201^Tl]Tl-noneunpa, and [^201^Tl]Tl-neunpa-NH_2_ in human serum were comparable after 1 h, with varying degrees of stability after 24 and 48 h. As none of the candidates were ideal with respect to stability, we based the selection of ligands for further evaluation on the ease of incorporation into bioconjugates.^28,30^ Additionally, small peptide imaging agents, such as PSMA-617, have very rapid blood clearance (<1 h), so prolonged complex stability (up to 24 or 48 h) is not essential but would be desirable. Thus, for a more detailed evaluation, we selected H_4_pypa, for which a PSMA-targeted peptide bioconjugate has recently been reported.^28^

The ^1^H NMR spectrum of the [^nat^Tl]Tl-pypa complex under mildly basic conditions could be interpreted as consistent with the presence of at least two non-interconverting (on the NMR time scale) species. This is not consistent with the HPLC data reported above for the [^201^Tl]Tl^3+^ complex, which may indicate that the HPLC method used was not capable of resolving multiple isomers/species. An alternative explanation is that in the acidic mobile phase used in HPLC analysis, interconversion between multiple species was rapid because of the dissociation of one or more carboxylate donor groups, which is suppressed under the basic conditions of ^1^H NMR but would have allowed the substitution of a carboxylate donor by water or an accessible dissociative mechanism of isomerization.

Crystals of the [Tl(Hpypa)] complex, enabling single crystal XRD analysis, were obtained from an acidic solution. The solid phase structure consists of a complex where one carboxylate group is pendant and protonated, with a Tl^3+^ coordination number of eight instead of the potential nine. This suggests that carboxylate group coordination is labile, and while this does not lead to the immediate dissociation or transchelation of Tl^3+^ in biological media, it might be expected to increase vulnerability to reduction by decreasing the coordination number and hence reducing electron density on the metal center. This is pertinent to the biological behavior of the complex bioconjugate, discussed below.

The PSMA-pypa conjugate was efficiently radiolabeled with ^201^Tl under conditions similar to those used for unconjugated H_4_pypa. The radiolabeled conjugate was biologically evaluated *in vitro* and *in vivo* using the prostate cancer cell line DU145 with and without PSMA expression. The *in vitro* data (Figure 5) indicate that in the presence of cells, ^201^Tl is released from the labeled bioconjugate complex, likely in the form of Tl^+^: the uptake of radioactivity in cells was initially low but increased with time, and over a period of an hour, the uptake pattern shifted to one that became similar to that of [^201^Tl]TlCl—that is, it reached levels similar to those typically observed for [^201^Tl]TlCl. ^201^Tl radioactivity uptake was similarly inhibited by potassium ions, was not selective for PSMA-positive cells, and was unaffected by a PSMA-specific blocking agent. This behavior can be interpreted on the basis that during the first few minutes of incubation, before the PSMA-specific binding of the radioconjugate has time to occur to a measurable extent, reducing agents secreted by cells into the medium prior to and after addition of the radioconjugate cause the reduction of [^201^Tl]Tl^3+^ to [^201^Tl]Tl^+^ and consequent release from the chelator. As this process develops over a period of minutes, the ^201^Tl radioactivity behaves biologically as Tl^+^ and is taken up efficiently by cells through the activity of the Na^+^/K^+^-ATPase pump, irrespective of PSMA expression.

This interpretation also accounts for the *in vivo* behavior as observed by SPECT imaging and *ex vivo* biodistribution. [^201^Tl]Tl^+^ shows the characteristic early myocardium uptake expected of a Na^+^/K^+^-ATPase substrate and myocardial imaging agent. This behavior is not greatly changed when the [^201^Tl]Tl^+^ is oxidized to [^201^Tl]Tl^3+^ before administration, consistent with the very rapid reduction upon initial exposure to the biological environment when unprotected by a Tl^3+^ chelator. The radiolabeled bioconjugate, on the other hand, shows a greatly reduced early uptake in the myocardium, indicating that the chelator survives and protects the Tl^3+^ from reduction and dissociation long enough to allow blood clearance (mainly via the kidney), potentially allowing the opportunity for modest selective uptake in PSMA-expressing tumors, as observed in the *in vivo* experiments on tumor-bearing mice. Although both suppression of myocardial uptake and a degree of PSMA-specific tumor uptake are observed, the tumor uptake is far below that required for effective imaging or treatment and is much less than is commonly observed with other PSMA-based tracers in this tumor model.^36^ The results are consistent with the hypothesis that dissociation is promoted by the reduction of the radiometal. This may well be facilitated by the acid-promoted release of a carboxylate donor, as observed in the X-ray crystal structure. The metal is left with reduced electron density and hence greater susceptibility to reduction.

## Conclusions

Seeking effective chelators for Tl^3+^, we have evaluated a series of polydentate N, O-ligands that have previously been shown to be effective chelators of other trivalent heavy metal ions often used in nuclear medicine. The findings indicate that the ligands form Tl^3+^ complexes more rapidly and efficiently than conventional chelators (DOTA, DTPA) and resist dissociation or transchelation in buffers free of biomolecules or reducing agents. In serum, however, dissociation occurs over several hours, albeit more slowly than is the case for DOTA and DTPA complexes.^23^ With H_4_pypa as an example studied in more detail, it became clear that bioreductive dissociation occurred much more quickly in the presence of living cells than in serum, leading to cellular uptake *in vitro* that was characteristic of [^201^Tl]TlCl. *In vivo*, a [^201^Tl]Tl-labeled pypa-PSMA conjugate possessed sufficient kinetic stability to show suppression of myocardial uptake and observable but practically inadequate selective delivery to PSMA-positive tumors. We conclude that the class of ligands studied here represents an advance on DOTA and DTPA but is not satisfactory as a basis for thallium-chelating bifunctional chelators. Further design improvement is needed, and this needs to take into account not only simple association/dissociation constants but also protection against reduction—by maximizing electron density donated to metal by maximizing the coordination number (by building in rigidity and preorganization) and incorporating more strongly electron donating donor groups.

## Materials and Methods

Unless stated otherwise, chemicals and solvents were purchased from commercial suppliers (Merck, Fisher Scientific, Fluorochem). H_4_noneunpa, H_5_decapa, and H_4_neunpa-NH_2_ were synthesized as reported.^24,41,42^ [^201^Tl]TlCl in saline was purchased from Curium Pharma, UK. Oxidation was performed using Pierce Iodination beads (Thermo Scientific). ^1^H NMR, ^13^C NMR, HSQC, and COSY data were acquired on a Bruker 400 MHz and analyzed using the MestReNova software. Flash chromatography purification was performed on a Biotage Isolera 4 flash chromatography system using Sfar chromatography columns (silica and C18). HPLC was performed on an Agilent 1260 Infinity instrument with UV spectroscopic detection at 254 nm and a Lablogic Flow-Count detector with a Bioscan Inc. B-FC-3200 photomultiplier tube detector and analyzed using the Lablogic Laura software. The mobile phase used for analytical and semipreparative reversed-phase HPLC was composed of (A) water with 0.1% TFA and (B) MeCN with 0.1% TFA. LC/MS data were acquired on an Agilent 1200 Series Liquid Chromatograph with UV spectroscopic detection at 254 nm and the same column details as in reversed-phase HPLC, interfaced with an Advion Expression LC/MS mass spectrometer with an electrospray ionization source. The mobile phase used for LC/MS was composed of (A) water with 0.1% formic acid and (B) MeCN with 0.1% formic acid using an Eclipse XDB-C18 column (4.6 × 150 mm, 5 μm). High-resolution electrospray mass spectrometry was carried out by Dr. Lisa Haigh (mass spectrometry service at Imperial College). Crystallographic data were collected using an Agilent Xcalibur PX Ultra A diffractometer, and the structures were refined using the SHELXTL^43^ and SHELX-2013^44^ program systems. Radioactive samples were measured using a Capintec CRC25R dose calibrator or an LKB Wallac 1282 Compugamma CS gamma counter for which data were collected using the EdenTerm software. SPECT/CT images were acquired using a NanoSPECT/CT scanner (Mediso Ltd., Budapest, Hungary) with 1.3 mm pinhole collimators and two energy windows at 72.3 keV ± 10% and 140.51 keV ± 10%. Images were reconstructed using the software package HiSPECT (ScivisGmbH) and analyzed using the VivoQuant software (version 3.5, InviCRO Inc.). Lyophilization was performed using an Edwards Freeze-Dryer Modulyo.

### Oxidation of [^201^Tl]Tl^+^ to [^201^Tl]Tl^3+^

This procedure is adapted from Rigby *et al*.^23^ [^201^Tl]TlCl (40 MBq, 100 μL) was added to one Pierce iodination bead in a 1.5 mL Eppendorf tube followed by the addition of HCl (0.5 M, 10 μL). The tube was vortexed, a small aliquot (2 μL) was removed for ITLC analysis, and the supernatant was pipetted into a clean tube. To measure the radiochemical conversion, acetone was used as the mobile phase and silica gel ITLC strips (iTLC-SG) were used as the stationary phase, giving good separation between [^201^Tl]Tl^+^ (*R*_f_ = 0) and [^201^Tl]Tl^3+^ (*R*_f_ = 1).

### Radiolabeling H_4_pypa, H_5_decapa, H_4_neunpa-NH_2_, and H_4_noneunpa with [^201^Tl]Tl^3+^

Chelators studied were H_4_pypa, H_5_decapa, H_4_neunpa-NH_2_, and H_4_noneunpa. The chelator solution (1 mg/mL in water, 20 μL) was added to [^201^Tl]TlCl_3_ (39.5 MBq, 108 μL) and 1 M ammonium acetate (pH 5, 20 μL). This was vortexed and agitated in a Thermomixer (500 rpm) at RT for 10 min. Radiochemical yield and purity were evaluated using RP-ITLC (unbound [^201^Tl]Tl^+^, [^201^Tl]Tl^3+^*R*_f_ = 0, [^201^Tl]Tl^3+^ complex *R*_f_ = 1) and HPLC (method 1). To measure radiochemical conversion, reversed-phase TLC plates (TLC Silica Gel 60 RP-18 F254s MS-grade) were used as the stationary phase, and acetonitrile (30%) with water was used as the mobile phase. All TLC plates were imaged using a Cyclone Plus Phosphor Imager (PerkinElmer, Inc. USA).

### Stability of [^201^Tl]Tl-pypa**,** [^201^Tl]Tl-decapa, [^201^Tl]Tl-neunpa-NH_2_, and [^201^Tl]Tl-noneunpa

Human serum (300 μL, Merck) was added to an Eppendorf tube followed by the addition of [^201^Tl]Tl-pypa, [^201^Tl]Tl-decapa, [^201^Tl]Tl-neunpa-NH_2_, or [^201^Tl]Tl-noneunpa (200 kBq, 12–15 μL). The tubes were then incubated at 37 °C for up to 48 h. Aliquots (2 μL) were removed at intervals and analyzed using RP-TLC to assess the stability. In addition to human serum, this process was repeated using an ammonium acetate solution (1 M, pH 5).

### Radiolabeling H_4_pypa-PSMA

A 1 mg/mL solution of H_4_pypa-PSMA was prepared in an ammonium acetate solution (1 M, pH 5). An aliquot of the H_4_pypa-PSMA solution (20 μL, 0.1 μM) was added to [^201^Tl]TlCl_3_ (110 MBq, 200 μL) followed by ammonium acetate (1 M, pH 5, 50 μL). This solution was vortexed and agitated in a Thermomixer (500 rpm) at RT for 10 min. Radiochemical yield and purity were evaluated using HPLC (method A, [^201^Tl]TlCl_3_*t*_R_ = 2.03 min; [^201^Tl]Tl-pypa-PSMA t_*R*_ = 15.02 min).

### Tissue Culture

DU145 (PSMA-negative) and DU145-PSMA (PSMA-positive) human prostate cancer cells were cultured in an RPMI-1640 medium supplemented with 10% fetal bovine serum, 2 mM l-glutamine, and penicillin/streptomycin (Sigma-Aldrich, UK) and maintained at 37 °C in a humidified atmosphere with 5% CO_2_.^45^ PSMA expression was evaluated using FACS, and the results can be found in Figure S1.

### SPECT Scanning and Biodistribution in Healthy and DU145-PSMA Tumor-Bearing Animals

Animal studies were carried out in accordance with the UK Home Office Animals (Scientific Procedures) Act 1986. Experiments complied with UK Research Councils’ and Medical Research Charities’ guidelines on responsibility in the use of animals in bioscience research under UK Home Office project and personal licenses. The reporting of this study complied with the Animal Research: Reporting In Vivo Experiments (ARRIVE) guidelines (https://www.nc3rs.org.uk/arrive-guidelines).

Healthy SCID/beige animals (male 5–7 weeks old, *n* = 3 per radiotracer) were injected via tail vein injection under isoflurane anesthesia (1.5–2.5% in oxygen at 1 L/min) with [^201^Tl]TlCl (17–22.9 MBq), [^201^Tl]TlCl_3_ (11.2–23.8 MBq), or [^201^Tl]Tl-pypa-PSMA (14.1–16.9 MBq)**.** Mice were then kept under continuous anesthesia on a heated pad for the duration of the experiment (1 h), and one mouse per group was imaged by SPECT/CT until 1 h post injection when animals were euthanized by cervical dislocation.

To study tracer uptake in tumors, SCID/beige mice (male 5–7 weeks old, *n* = 3 per group) were injected subcutaneously with DU145-PSMA or DU145 cells (4 × 10^6^ cells in 100 mL PBS) in the left shoulder. Once tumors had reached 5–10 mm in diameter (4–5 weeks after inoculation), [^201^Tl]Tl-pypa-PSMA (10.7–24.5 MBq, 20 mmol) was administered via tail vein injection under isoflurane anesthesia. Mice were maintained under continuous anesthesia and imaged by SPECT/CT for up to 2 h post injection. Animals were then euthanized by cervical dislocation. SPECT images were reconstructed using the HiSPECT (Scivis GmbH) reconstruction software package at 0.3 mm isotropic voxel size using standard reconstruction with 35% smoothing and nine iterations. After euthanasia, organs were harvested from the mice, weighed, and gamma counted.

### Image Analysis

Images were analyzed using VivoQuant 2.5 (InviCRO LLC., Boston, USA), enabling the delineation of regions of interest (ROIs) for quantification of radioactivity. ROIs for the tumor and organs (heart, muscle, etc.) were drawn using CT images, and volumes were determined. The total activity in the whole animal (excluding the majority of tail, out of SPECT field of view) at the time of [^201^Tl] agents’ administration was defined as the injected activity (IA), and the percentage of injected activity per cm^3^ (% IA/cm^3^) and amount of radioactivity in tissues (MBq) were determined. A 5 mL syringe with 3 mL of [^201^Tl]TlCl (40 MBq) was used to calibrate the SPECT/CT and ensure correct co-registration between the SPECT and CT.

### Statistical Analysis

Data are reported as average ± standard deviation. Statistical analysis was performed using Graphpad Prism Version 7.0c with unpaired *t* tests used in uptake and a two-way ANOVA with Sidak’s multiple comparisons test used for *in vivo* studies; *p ≤ 0.05, **p ≤ 0.01, ***p ≤ 0.001, and ****p ≤ 0.0001.

### Synthesis

#### Di-*tert*-butyl (1*H*-Imidazole-1-carbonyl)glutamate (**1**)

**1** was synthesized using a previously reported method by Duspara *et al*.^35^l-Glutamic acid di-*tert*-butyl hydrochloride (3.56 g, 12.04 mmol) and carbonyldiimidazole (2.15 g, 13.24 mmol) were dissolved in a 1:5 mixture of DMF/MeCN (50 mL) and stirred at RT overnight. MeCN was then removed *in vacuo*, and the remaining DMF was diluted with EtOAc (100 mL) and washed with water (3 × 50 mL) and brine (3 × 50 mL). The organic layer was then dried over magnesium sulfate, and the solvent was removed *in vacuo*. The crude product was then purified using a Biotage Isolera flash chromatography system (20–80% EtOAc/petroleum ether) to yield the desired product as a colorless oil that solidified upon standing (2.1 g, 51%). ^1^H NMR (400 MHz, chloroform-*d*) δ 8.16 (t, *J* = 1.1 Hz, 1H), 7.57 (d, *J* = 6.8 Hz, 1H), 7.41 (t, *J* = 1.5 Hz, 1H), 7.07 (dd, *J* = 1.6, 0.9 Hz, 1H), 2.48–2.38 (m, 2H), 2.27–2.05 (m, 2H), 1.47 (s, 9H), 1.43 (s, 9H). ^13^C NMR (101 MHz, chloroform-*d*) δ 174.00, 173.50, 173.34, 171.69, 162.78, 157.36, 149.11, 136.24, 135.17, 129.96, 121.71, 116.40, 77.43, 77.11, 76.79, 53.49, 52.72, 52.53, 52.38, 52.35, 52.12, 51.83, 51.78, 36.61, 31.51, 30.33, 30.07, 27.98, 27.94, 27.78, 26.15. ESI-MS: calc. for [C_17_H_27_N_3_O_5_ + H]^+^ 354.42; found 354.35.

#### Tri-*tert*-butyl 3,11-Dioxo-1-phenyl-2-oxa-4,10,12-triazapentadecane-9,13,15-tricarboxylate (**2**)

**2** was synthesized by adapting a previously reported method by Duspara *et al*.^35^ H-Lys(Z)-OtBu·HCl (3.47 g, 9.34 mmol) was dissolved in DMF (20 mL). DIPEA (1.63 mL, 9.34 mmol) was added to the solution followed by **1** (3 g, 8.49 mmol, dissolved in 10 mL DMF) dropwise and allowed to stir overnight at RT. The reaction was diluted with EtOAc (100 mL) and washed with water (3 × 100 mL) and brine (3 × 100 mL). The organic layer was then dried over magnesium sulfate, and the solvent was removed *in vacuo*. The crude product was purified using a Biotage Isolera flash chromatography system (20–80% EtOAc/petroleum ether) to yield the desired product as a colorless oil (4.5 g, 83%). ^1^H NMR (400 MHz, chloroform-*d*) δ 7.38–7.30 (m, 5H), 5.22–5.02 (m, 5H), 4.33 (dd, *J* = 8.1, 4.9 Hz, 2H), 3.17 (dd, *J* = 6.4, 3.7 Hz, 2H), 2.28 (td, *J* = 9.6, 6.4 Hz, 2H), 1.44 (d, *J* = 1.1 Hz, 18H), 1.43 (s, 10H). ^13^C NMR (101 MHz, chloroform-*d*) δ 172.41, 156.85, 156.59, 136.71, 128.46, 128.05, 128.00, 82.10, 81.75, 80.51, 77.33, 77.02, 76.70, 66.55, 53.29, 53.02, 40.65, 32.65, 31.60, 29.36, 28.36, 28.08, 28.03, 28.00, 22.24. ESI-MS: calc. for [C_32_H_51_N_3_O_9_ + H]^+^ 622.36; found 622.3.

#### Di-*tert*-butyl ((6-Amino-1-(tert-butoxy)-1-oxohexan-2-yl)carbamoyl)glutamate (**3**)

The cbz protected urea **2** (3.6 g, 5.79 mmol) was dissolved in methanol (20 mL) and added to Pd/C (10%, 0.125 g, 1.16 mmol). The reaction flask was evacuated before being flushed with two balloons of hydrogen gas and a third balloon left connected to the vessel for the duration of the experiment. TLC analysis of the reaction showed completion after 90 min. The Pd/C was removed via filtration through Celite, and the solvent was removed *in vacuo* to yield a colorless oil. This was then purified using the Biotage Isolera flash chromatography system (reversed-phase SFar C18 column, 0–60% MeCN/0.1% FA:H_2_O/0.1% FA) to yield the desired product as a colorless oil that solidified under a vacuum (2.62 g, 92%). ^1^H NMR (400 MHz, chloroform-*d*) δ 6.37 (d, *J* = 8.1 Hz, 1H), 6.23 (d, *J* = 8.0 Hz, 1H), 4.31 (s, 2H), 2.98 (s, 2H), 2.32 (dd, *J* = 6.5, 3.2 Hz, 2H), 1.71 (s, 4H), 1.44 (d, *J* = 1.8 Hz, 18H), 1.43 (s, 10H). ^13^C NMR (101 MHz, chloroform-*d*) δ 173.62, 172.80, 172.36, 157.65, 82.11, 81.54, 80.53, 77.33, 77.01, 76.70, 53.12, 52.88, 39.20, 31.78, 31.28, 28.10, 28.04, 27.20, 21.68. ESI-MS: calc. for [C_24_H_45_N_3_O_7_ + H]^+^ 488.64; found 488.45.

#### Di-*tert*-butyl ((6-(2-(((Benzyloxy)carbonyl)amino)-3-(naphthalen-2-yl)propanamido)-1-(*tert*-butoxy)-1-oxohexan-2-yl)carbamoyl)glutamate (**4**)

Z-3-(2-naphthyl)-d-alanine (0.395 g, 1.13 mmol) and HATU (0.858 g, 2.26 mmol) were dissolved in dry DMF (10 mL) followed by the addition of DIPEA (0.54 mL, 3.08 mmol), with the solution turning from colorless to yellow. This was left to stir for 15 min at RT, after which **3** (0.5 g, 1.03 mmol), dissolved in dry DMF (5 mL), was added to the stirring solution and left at RT to stir overnight. During this time, the reaction had turned dark brown in color. The reaction was diluted with EtOAc (100 mL) and washed with water (3 × 50 mL) and brine (3 × 50 mL). The organic layer was then dried over magnesium sulfate, and the solvent was removed *in vacuo*. The crude product was then purified using a Biotage Isolera flash chromatography system (20–70% EtOAc/petroleum ether) to yield the desired product as a yellow oil (0.46 g, 55%). ^1^H NMR (400 MHz, chloroform-*d*) δ 7.83 (s, 3H), 7.77 (s, 1H), 7.71 (d, *J* = 8.2 Hz, 3H), 7.66 (s, 2H), 7.59 (d, *J* = 10.9 Hz, 3H), 7.51–7.33 (m, 4H), 7.27 (d, *J* = 11.8 Hz, 4H), 7.22–7.11 (m, 1H), 5.09 (s, 5H), 4.96 (d, *J* = 13.0 Hz, 1H), 4.35 (s, 1H), 4.12 (q, *J* = 7.1 Hz, 1H), 3.43 (s, 1H), 3.23 (s, 1H), 3.19–3.03 (m, 1H), 3.03–2.73 (m, 1H), 1.93–1.64 (m, 2H), 1.44 (d, *J* = 2.5 Hz, 20H), 1.41 (s, 9H). ^13^C NMR (101 MHz, chloroform-*d*) δ 133.47, 132.36, 128.52, 128.26, 128.14, 127.67, 126.23, 80.57, 77.34, 77.02, 76.70, 60.39, 53.41, 53.11, 31.83, 28.11, 28.02, 21.04, 14.20. HR-ESI-MS: calc. for [C_45_H_62_N_4_O_10_ + H]^+^ 819.4544; found 819.4550.

#### Di-*tert*-butyl ((6-(2-Amino-3-(naphthalen-2-yl)propanamido)-1-(*tert*-butoxy)-1-oxohexan-2-yl)carbamoyl)glutamate (**5**)

**4** (0.185 g, 0.24 mmol) was dissolved in methanol (10 mL) and added to Pd/C (10%, 0.007 g, 0.05 mmol). The reaction flask was evacuated before being flushed with two balloons of hydrogen gas and a third balloon left connected to the vessel for the duration of the experiment. TLC analysis of the reaction showed completion after stirring overnight. The Pd/C was removed via filtration through Celite, and the solvent was removed *in vacuo* to yield a pale-yellow oil (0.149 g, 89%). ^1^H NMR (400 MHz, chloroform-*d*) δ 7.84–7.73 (m, 3H), 7.67 (s, 1H), 7.57 (s, 1H), 7.47–7.41 (m, 2H), 7.35 (d, *J* = 8.2 Hz, 1H), 4.29 (s, 1H), 4.19 (s, 2H), 3.32 (s, 1H), 3.20 (s, 1H), 3.08 (s, 2H), 2.38–2.19 (m, 2H), 2.13–1.95 (m, 1H), 1.91–1.78 (m, 1H), 1.74 (t, *J* = 3.3 Hz, 2H), 1.41 (s, 10H), 1.40 (d, *J* = 1.6 Hz, 18H). ^13^C NMR (101 MHz, chloroform-*d*) δ 172.93, 172.83, 172.50, 170.65, 157.44, 133.44, 133.20, 132.53, 128.53, 128.40, 127.68, 127.64, 127.29, 126.28, 125.91, 82.13, 81.60, 80.62, 77.35, 77.03, 76.72, 55.40, 53.43, 52.96, 39.02, 38.65, 31.71, 28.59, 28.28, 28.06, 27.99, 27.97, 22.00. HR-ESI-MS: calc. for [C_37_H_56_N_4_O_8_ + H]^+^ 685.4176; found 685.4188.

#### Di-*tert*-butyl ((6-(2-(4-((((Benzyloxy)carbonyl)amino)methyl)cyclohexane-1-carboxamido)-3-(naphthalen-2-yl)propanamido)-1-(*tert*-butoxy)-1-oxohexan-2-yl)carbamoyl)glutamate (**6**)

Trans-4-(cbz-amino)cyclohexanecarboxylic acid (0.153 g, 0.53 mmol) and HATU (0.852 g, 1.1 mmol) were dissolved in dry DMF followed by the addition of DIPEA (0.16 mL, 1.56 mmol), with the solution turning from colorless to yellow. This was left to stir for 15 min at RT, after which **5** (0.3 g, 0.44 mmol), dissolved in dry DMF (5 mL), was added dropwise to the stirring solution and left to stir overnight. During this time, the reaction turned dark brown in color. The reaction was diluted with EtOAc (100 mL) and washed with water (3 × 50 mL) and brine (3 × 50 mL). The organic layer was then dried over magnesium sulfate, and the solvent was removed *in vacuo*. The crude product was then purified using a Biotage Isolera flash chromatography system (20–70% EtOAc/petroleum ether) to yield the desired product as a yellow oil (0.24 g, 63%). ^1^H NMR (400 MHz, chloroform-*d*) δ 7.89–7.78 (m, 1H), 7.73 (t, *J* = 6.9 Hz, 2H), 7.63 (d, *J* = 1.6 Hz, 1H), 7.52–7.42 (m, 2H), 7.34 (d, *J* = 4.6 Hz, 6H), 5.26 (d, *J* = 7.7 Hz, 1H), 5.20 (d, *J* = 8.3 Hz, 1H), 4.90 (s, 1H), 4.71 (d, *J* = 7.4 Hz, 1H), 4.37–4.26 (m, 1H), 4.14 (q, *J* = 4.1 Hz, 1H), 3.39–3.26 (m, 1H), 3.20 (d, *J* = 6.4 Hz, 1H), 3.17–3.07 (m, 4H), 3.02 (t, *J* = 6.4 Hz, 2H), 2.32 (td, *J* = 9.5, 6.4 Hz, 2H), 2.06 (tdd, *J* = 11.3, 5.2, 3.1 Hz, 2H), 1.89–1.72 (m, 8H), 1.70–1.61 (m, 2H), 1.44 (d, *J* = 1.5 Hz, 18H), 1.42 (s, 10H). ^13^C NMR (101 MHz, chloroform-*d*) δ 175.84, 172.80, 172.51, 172.46, 171.54, 157.03, 136.59, 134.54, 133.41, 132.32, 128.51, 128.12, 127.62, 126.23, 125.79, 82.11, 81.46, 80.63, 77.34, 77.03, 76.71, 66.68, 54.79, 53.23, 52.94, 45.02, 39.16, 38.39, 37.55, 31.78, 31.66, 29.67, 28.92, 28.74, 28.59, 28.51, 28.11, 28.03, 21.87. HR-ESI-MS: calc. for [C_53_H_75_N_5_O_11_ + H]^+^ 958.5541; found 958.5559.

#### Di-*tert*-butyl ((6-(2-(4-(Aminomethyl)cyclohexane-1-carboxamido)-3-(naphthalen-2-yl)propanamido)-1-(*tert*-butoxy)-1-oxohexan-2-yl)carbamoyl)glutamate (**7**)

**6** (0.081 g, 0.83 mmol) was dissolved in dry MeOH (10 mL) and Pd/C (10%, 0.003 g, 0.016 mmol) added. The reaction flask was evacuated before being flushed with two balloons of hydrogen gas and a third balloon left in the vessel for the duration of the experiment. TLC analysis of the reaction showed completion after stirring overnight. The Pd/C was removed via filtration through Celite, and the solvent was removed *in vacuo* to yield **7** as a yellow oil (0.062 g, 91%). ^1^H NMR (400 MHz, chloroform-*d*) δ 7.77–7.67 (m, 3H), 7.65 (s, 1H), 7.42–7.31 (m, 3H), 5.75 (d, *J* = 18.6 Hz, 2H), 4.74 (s, 1H), 4.28 (d, *J* = 6.6 Hz, 1H), 4.08 (d, *J* = 6.4 Hz, 1H), 3.17 (d, *J* = 10.3 Hz, 2H), 3.06 (s, 2H), 2.72 (s, 3H), 2.31 (q, *J* = 7.0, 6.3 Hz, 3H), 2.16–1.98 (m, 4H), 1.92–1.81 (m, 2H), 1.69 (s, 4H), 1.43 (d, *J* = 1.7 Hz, 18H), 1.41 (s, 9H). HR-ESI-MS: calc. for [C_45_H_69_N_5_O_9_ + H]^+^ 824.5168; found 824.5174.

#### 6,6′-((((4-(4-(3-((4-((7,11-Bis(*tert*-butoxycarbonyl)-2,2-dimethyl-19-(naphthalen-2-yl)-4,9,17-trioxo-3-oxa-8,10,16-triazanonadecan-18-yl)carbamoyl)cyclohexyl)methyl)thioureido)phenethoxy)pyridine-2,6-diyl)bis(methylene))bis((carboxymethyl)azanediyl))bis(methylene))dipicolinic Acid (**26**)

**20** (0.01 g, 14.3 μmol) was dissolved in CHCl_3_ (1 mL). **7** (0.012 g, 14.3 μmol) was separately dissolved in CHCl_3_ (1 mL). Triethylamine (2 × 4 μL, 57 μmol) was added to each solution, and then both solutions were mixed. This was allowed to stir at RT overnight, after which the CHCl_3_ was removed *in vacuo*. The product was then purified using reversed-phase semipreparative HPLC (A: MeCN/0.1% TFA, B: H_2_O/0.1% TFA, 5–80% A over 60 min, 4 mL/min). UV-active fractions were analyzed using LC–MS (HPLC method B); pure fractions were combined and freeze-dried to yield the product as a white solid (0.012 g, 56%) ESI-MS: calc. for [C_79_H_101_N_11_O_18_S + H]^+^ 1525.80; found 1525.04.

#### ((5-(2-(4-((3-(4-(2-((2,6-Bis(((carboxymethyl)((6-carboxypyridin-2-yl)methyl)amino)methyl)pyridin-4-yl)oxy)ethyl)phenyl)thioureido)methyl)cyclohexane-1-carboxamido)-3-(naphthalen-2-yl)propanamido)-1-carboxypentyl)carbamoyl)glutamic Acid (**27**)

**21** (0.01 g, 6.6 μmol) was dissolved in DCM/TFA (1:1, 4 mL) and allowed to stir at RT overnight. The solution was then concentrated *in vacuo*, and the residue was redissolved in deionized water and purified using reversed-phase semipreparative HPLC (A: MeCN/0.1% TFA, B: H_2_O/0.1% TFA, 5–80% A over 60 min, 4 mL/min). UV-active fractions were analyzed using LC–MS (HPLC method B); pure fractions were combined and freeze dried to yield the product as a white solid (0.006 g, 75%). HR-ESI-MS: calc. for [C_67_H_77_N_11_O_18_S + H]^+^ 1356.5247; found 1356.5298.

### [^nat^Tl]Tl-pypa

TlCl_3_ hydrate (0.05 g, 96 μmol) was dissolved in ammonium acetate solution (1 M, pH 5, 0.5 mL) and added to a solution of H_4_pypa (**15**) (0.037 g, 96 μmol) also dissolved in ammonium acetate solution (1 M, pH 5, 0.5 mL). The mixture was agitated for 5 min at RT, and an aliquot was removed for analysis using LC–MS. The complex was purified using reversed-phase preparative HPLC (A: MeCN/0.1% TFA, B: H_2_O/0.1% TFA, 5–60% A over 40 min, 10 mL/min). UV-active fractions were analyzed using LC–MS (HPLC method B); pure fractions were combined and freeze-dried to yield the product as a white solid (0.055 g, 80%) HR-ESI-MS: calc. for [C_25_H_23_N_5_O_8_^205^Tl + H]^+^ 726.1291; found 726.1306.

## Supporting Information Available

NMR (^1^H, ^13^C, COSY, HSQC) spectra for compounds **1**–**7** and [^nat^Tl]Tl-pypa; HR-MS spectra for compounds **1**–**7** and [^nat^Tl]Tl-pypa; X-ray crystallographic data for [^nat^Tl] [Tl(Hpypa)]; validation of PSMA expression in the cells used; description of the HPLC methods used, as well as the [^201^Tl]TlCl and [^201^Tl]TlCl_3_ HPLC traces and the reactions between [^201^Tl]TlCl with each chelator; further stability data against transmetalation in excess transferrin and in the buffer for all four [^201^Tl]Tl-complexes; analytical HPLC trace for H_4_pypa-PSMA; and additional information on [^201^Tl]Tl-pypa-PSMA cell uptake experiments (PDF)
