# Supplementary material for: Toward Bifunctional Chelators for Thallium-201 for Use in Nuclear Medicine
Source: Bioconjug Chem. 2022 Jul 8;33(7):1422–36. doi: 10.1021/acs.bioconjchem.2c00284 (PMC9305974; doi:10.1021/acs.bioconjchem.2c00284)
Supplement: Supplementary file 1 — bc2c00284_si_001.pdf [file bc2c00284_si_001.pdf]

Supporting information

Towards bifunctional chelators for thallium-201 for use in nuclear medicine

Alex Rigby[1], George Firth[1], Charlotte Rivas[1], Truc Pham[1], Jana Kim[1], Andreas Phanopoulos[2], Luke Wharton[3,4], Aidan Ingham[3,4], Lily Li[3,4], Michelle T Ma[1], Chris Orvig[3], Philip J Blower[1], Samantha Y A Terry[1] and Vincenzo Abbate[5*]*

[1] King's College London, School of Biomedical Engineering and Imaging Sciences, 4th Floor Lambeth Wing, St Thomas' Hospital, London, SE1 7EH, United Kingdom

[2] Department of Chemistry, Imperial College London, Molecular Sciences Research Hub, London, W12 0BZ, United Kingdom

[3] Medicinal Inorganic Chemistry Group, Department of Chemistry, University of British Columbia, Vancouver, BC V6T 1Z1, Canada

[4] Life Sciences Division, TRIUMF, 4004 Wesbrook Mall, Vancouver, BC V6T 2A3, Canada

[5] King's College London, School of Cancer & Pharmaceutical Sciences, Franklin-Wilkins Building, Stamford Street, London, SE1 9NH, United Kingdom

* Contributed equally as last authors

Corresponding authors: Dr Samantha YA Terry, King's College London, School of Biomedical Engineering & Imaging Sciences, 4th floor Lambeth Wing, St Thomas' Hospital, London, SE1 7EH. United Kingdom.

samantha.terry@kcl.ac.uk, +44 2071887188 ext. 85438.

Dr Vincenzo Abbate, King's College London, School of Cancer & Pharmaceutical Sciences, Franklin-Wilkins Building, Stamford Street, London, SE1 9NH, United Kingdom

vincenzo.abbate@kcl.ac.uk, +44 20784884489

Table of Contents

Validating PSMA expression in DU145 PSMA positive and negative cell lines 3

High performance liquid chromatography (HPLC) methods 4

HPLC controls of [^{201}Tl]TlCl and [^{201}Tl]$TlCl_3$ 5

HPLC controls of [^{201}Tl]TlCl with each chelator 6

Stability studies 7

HPLC traces 11

Uptake of [^{201}Tl]Tl-pypa-PSMA in DU145 PSMA positive and negative cells 11

High resolution mass spectra 22

X-ray crystallography 25

Validating PSMA expression in DU145 PSMA positive and negative cell lines

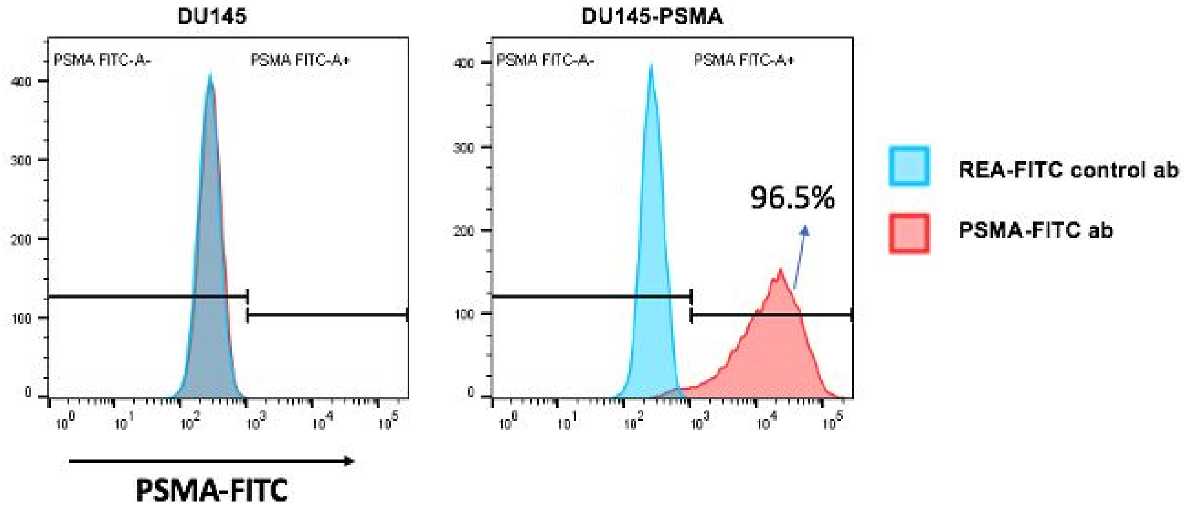

Figure S1 – FACS results showing the expression of the PSMA receptor of the DU145 PSMA positive and negative cells used in these experiments

Expression of GCP(II)/PSMA in the DU145-PSMA cell line and the absence of this receptor in the DU145 cell line was assessed using flow cytometry. 1 × 10^6 cells were suspended in 100 µL PBS containing 0.5% bovine serum albumin and then incubated with 2 µL of either Anti-PSMA anti-human VioBright-FITC antibody (Miltenyi Biotec, Clone REA408) or REA-control human IgG1 VioBright-FITC (Miltenyi Biotec, Clone REA293), for 15 minutes on ice. After this time the cells were washed twice with PBS and then analysed on a BD FACSMelody™. Gating and analysis were performed using FlowJo™ software (BD, v.10.8).

High performance liquid chromatography (HPLC) methods

HPLC method A. Solvent A = water (+ 0.1% TFA), solvent B = acetonitrile (+ 0.1% TFA). Column = Agilent Eclipse XDB-C18 column (4.6 x 150 mm, 5 uM) analytical.

Time (minutes)	Flow rate (mL min^{-1})	% solvent A	% solvent B
0	1	100	0
2	1	100	0
25	1	5	95
25.1	1	100	0
30	1	100	0

HPLC method B. Solvent A = water (+ 0.1% TFA), solvent B = acetonitrile (+ 0.1% TFA). Column = Agilent Eclipse XDB-C18 column (4.6 x 150 mm, 5 uM) analytical.

Time (minutes)	Flow rate (mL min^{-1})	% solvent A	% solvent B
0	1	95	5
2	1	95	5
11	1	5	95
12	1	5	95
12.1	1	95	5
15	1	95	5

HPLC controls of [^{201}Tl]TlCl and [^{201}Tl]TlCl$_3$

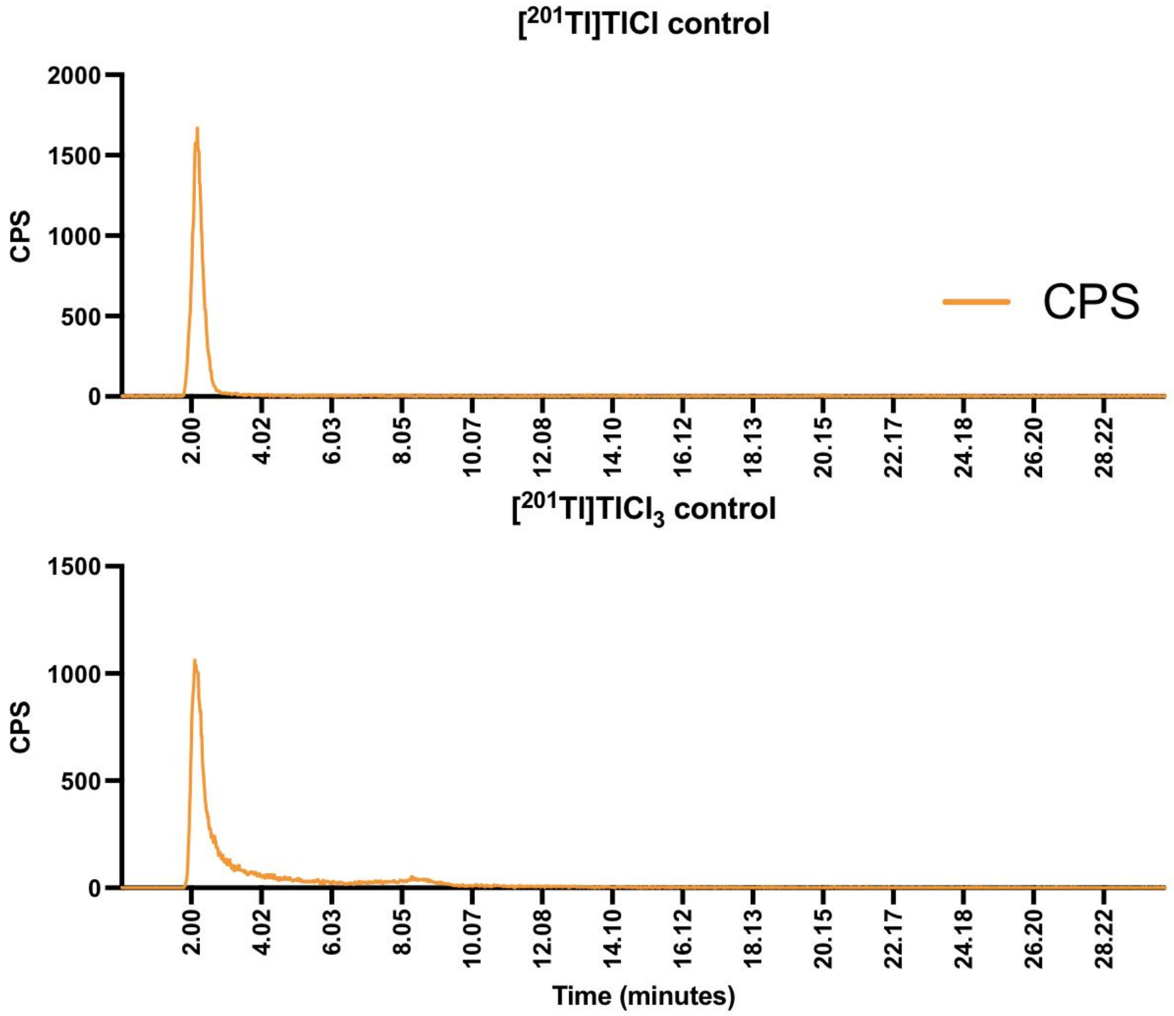

Figure S2 - Analytical HPLC trace of [^{201}Tl]TlCl (top) and [^{201}Tl]TlCl$_3$ (bottom) using HPLC method A (orange = counts per second) (HPLC method A).

HPLC controls of [^{201}Tl]TlCl with each chelator

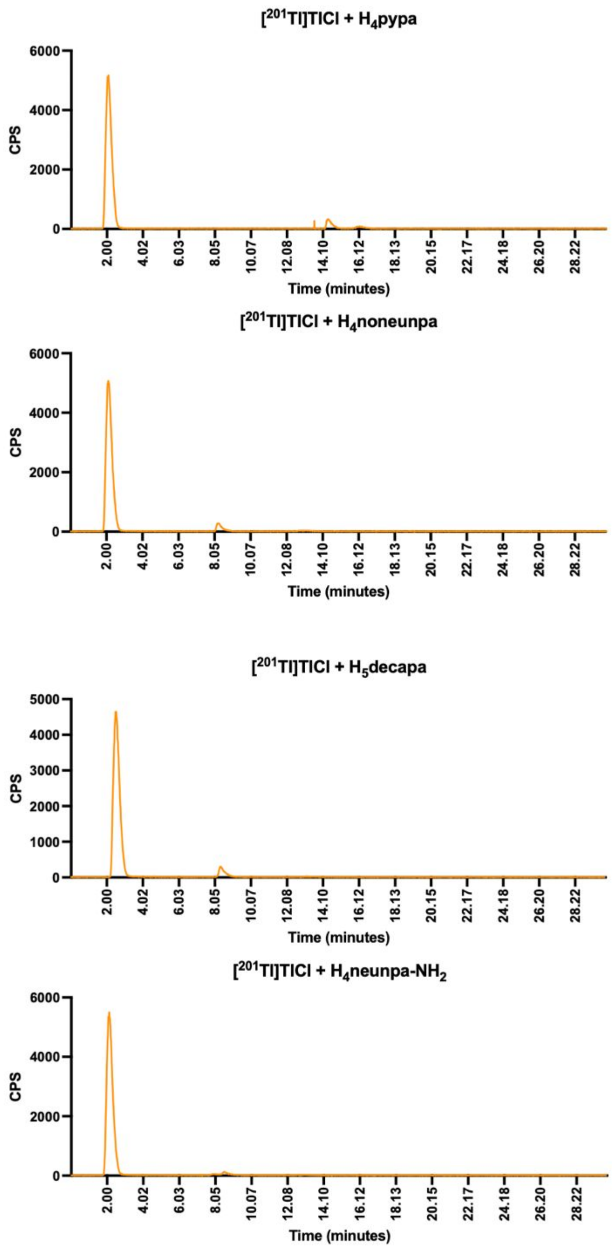

Figure S3 - Analytical HPLC trace of [^{201}Tl]TlCl reacted with each of the chelators (H_4pypa, H_5decapa, H_4noneunpa, and H_4neunpa-NH_2) using HPLC method A (orange = counts per second) (HPLC method A).

Stability studies

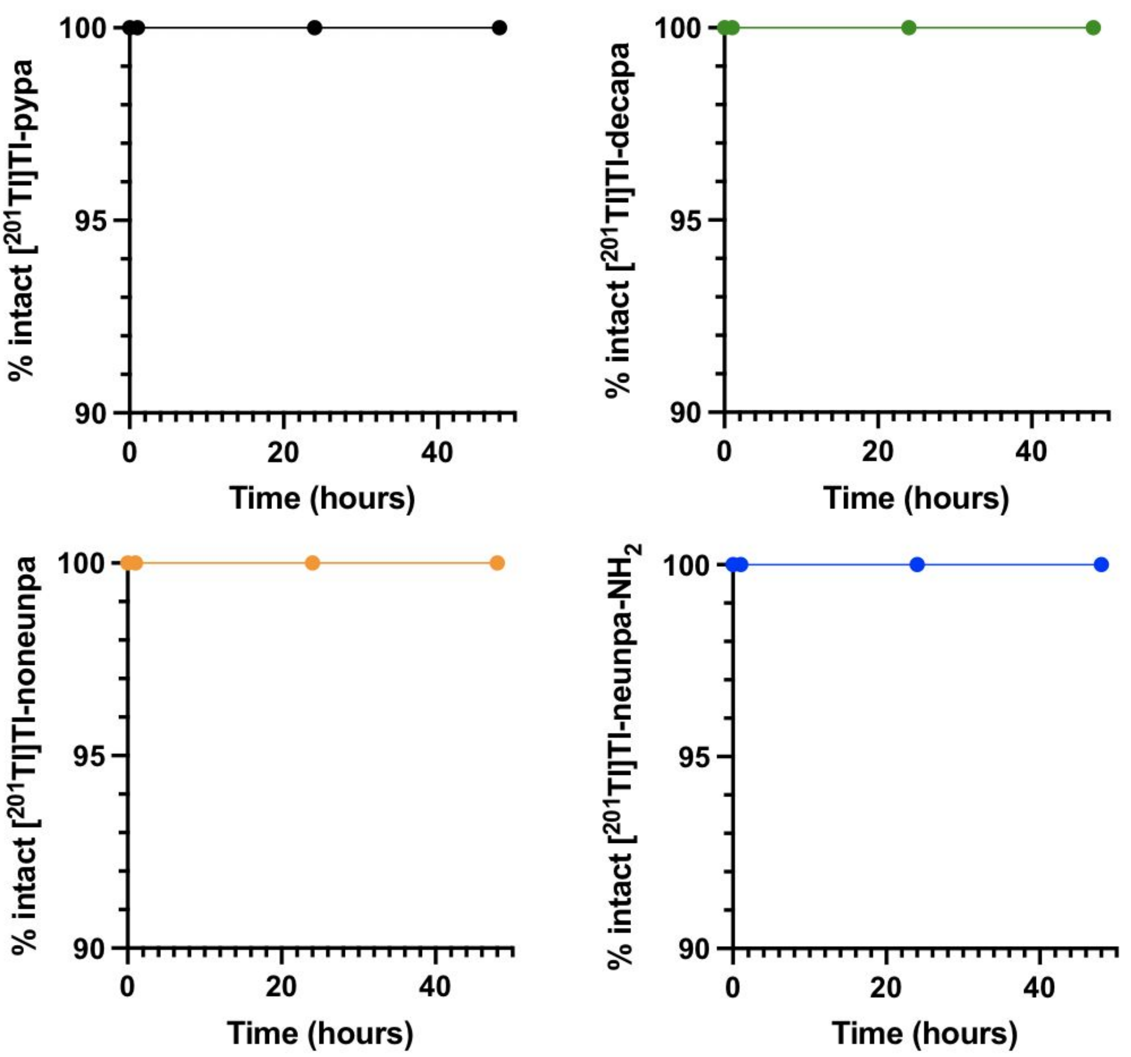

Figure S4 – Stability studies in ammonium acetate solution (1 M, pH 5) for [^{201}Tl]Tl-pypa, [^{201}Tl]Tl-decapa, [^{201}Tl]Tl-neunpa-NH_2 and [^{201}Tl]Tl-noneunpa (n=2/3).

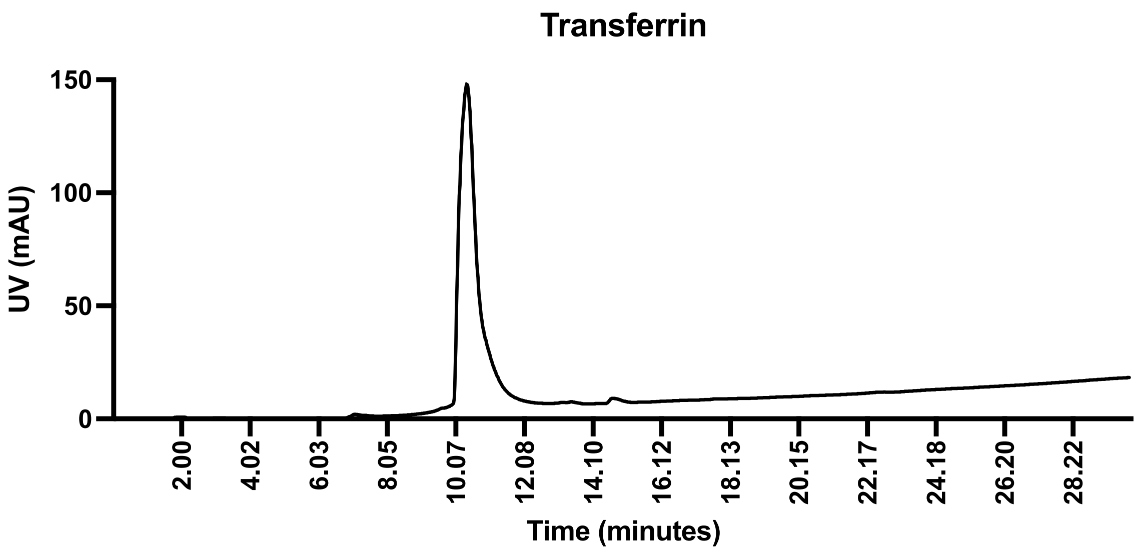

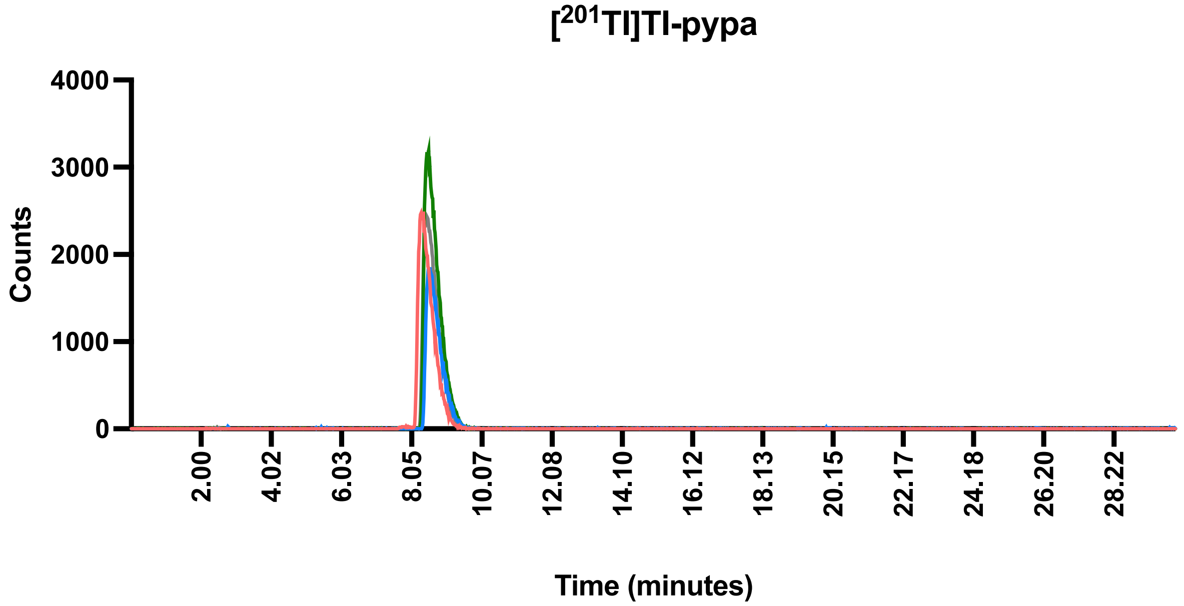

Figure S5 – Top - Analytical HPLC trace of transferrin. Bottom – Analytical HPLC trace of [^{201}Tl]Tl-pypa following incubation with excess transferrin using HPLC method A (orange = 0h, blue = 1h, green = 4h, grey = 24h) (HPLC method A).

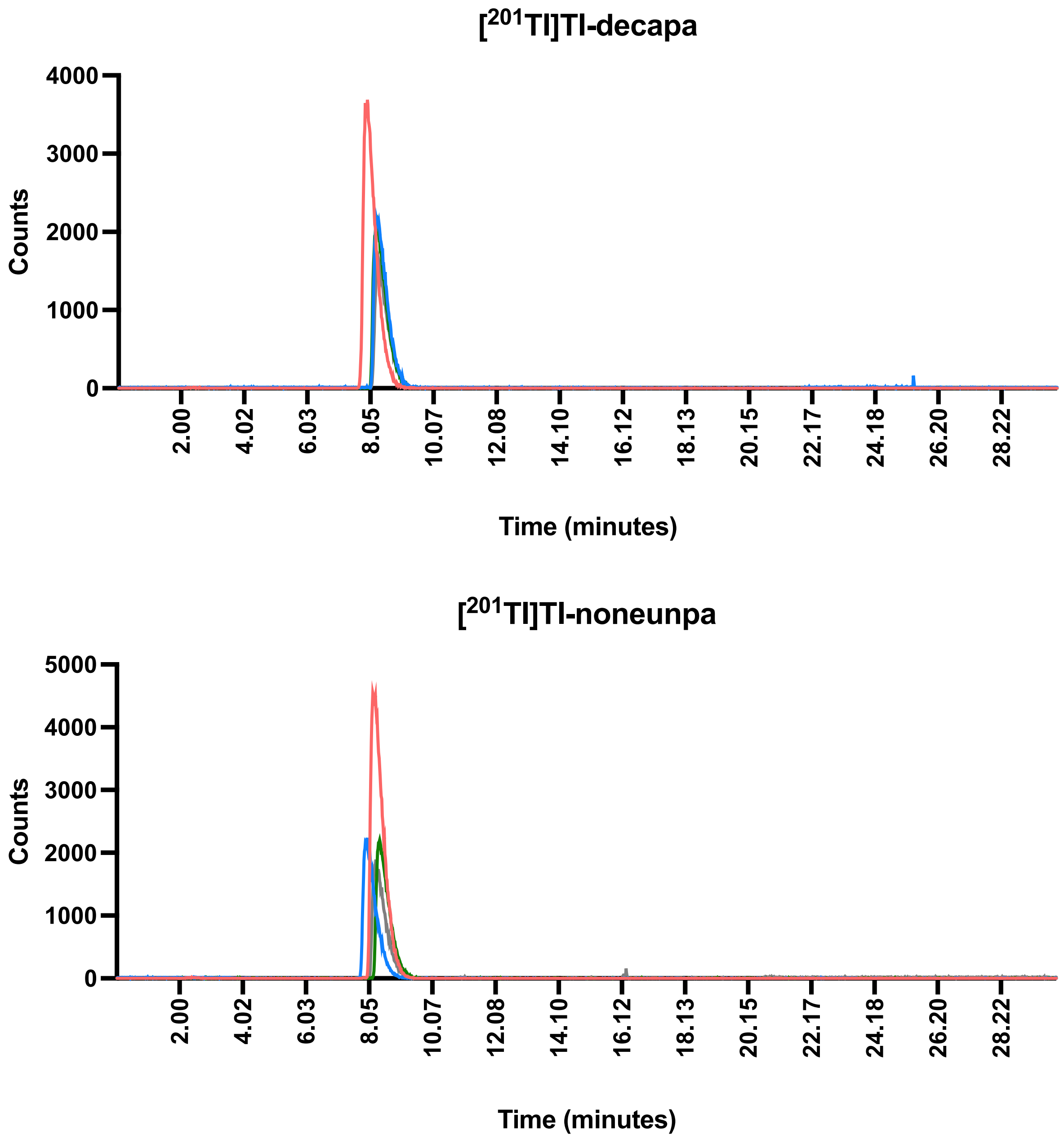

Figure S6 - Top - Analytical HPLC trace of [^{201}Tl]Tl-decapa following incubation with excess transferrin using HPLC method A. Bottom – Analytical HPLC trace of [^{201}Tl]Tl-noneunpa following incubation with excess transferrin using HPLC method A (orange = 0h, blue = 1h, green = 4h, grey = 24h) (HPLC method A).

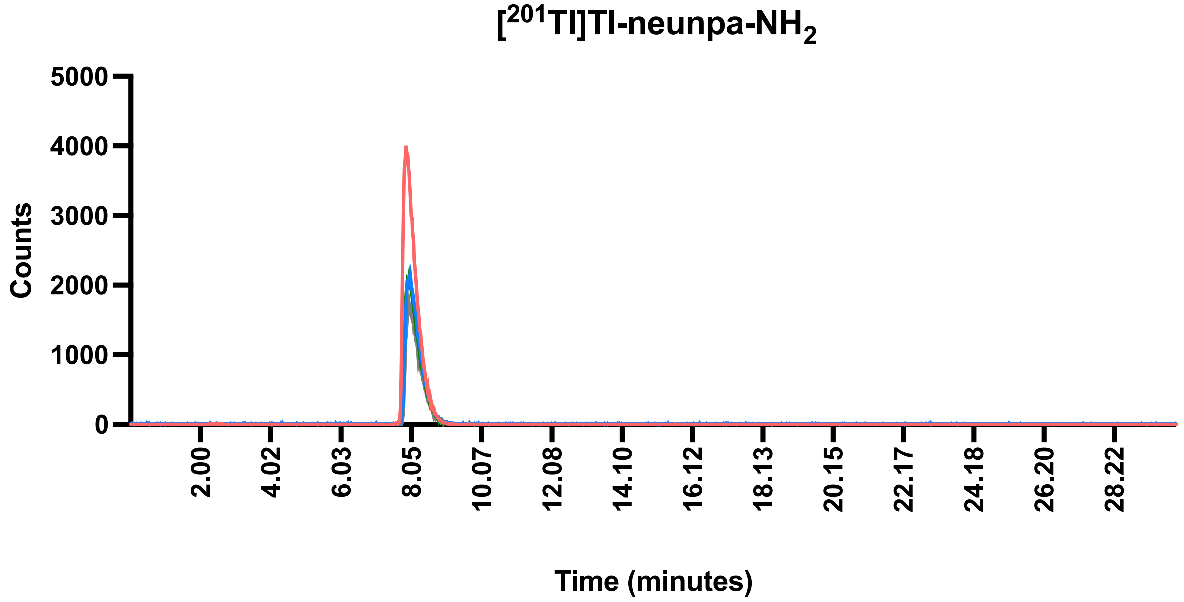

Figure S7 - Analytical HPLC trace of [^{201}Tl]Tl-neunpa-NH$_2$ following incubation with excess transferrin using HPLC method A.

HPLC traces

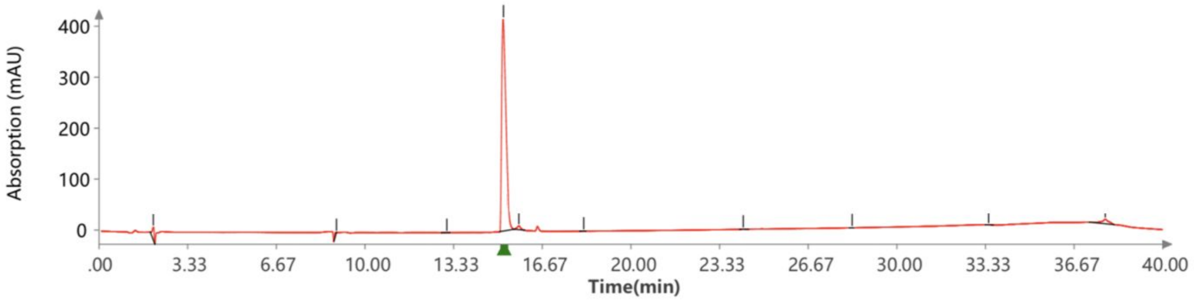

Figure S8 -HPLC-UV (254 nm) chromatogram of unlabelled H_4pypa-PSMA.

Uptake of [^{201}Tl]Tl-pypa-PSMA in DU145 PSMA positive and negative cells

DU145 (PSMA-negative) and DU145-PSMA (PSMA-positive) human prostate cancer cells were cultured in RPMI 1640 medium (R0883, Sigma) supplemented with 10% (v/v) foetal bovine serum (FBS), 2 mM L-glutamine, and penicillin/streptomycin (Sigma-Aldrich, UK) and maintained in a humidified atmosphere at 37 ˚C under 5% CO_2.

To assess PSMA targeting, PSMA-expressing cells DU145-PSMA and non-PSMA-expressing cells DU145 cells were seeded in 6-well plates at a density of 0.5 × 10 cells/well 1 day prior to the experiment. Three technical replicates were performed for each condition. Cell medium was replaced with 1 mL complete medium 1 hour before the cells were treated. [^{201}Tl]Tl-pypa-PSMA (100 kBq, in 10 µL of ammonium acetate buffer (1M)) was added to each well, and the cells incubated at 37 ˚C for 15 min and 60 minutes. Competition studies were also performed following co-incubation with the PSMA-inhibitor 2-(phosphonomethyl)pentane-1,5-dioic acid (PMPA; 30 µL of 750 mM PMPA solution/well). After incubation, plates were placed on ice, the supernatant was removed, and the cells were washed with ice cold phosphate buffered saline solution (2 × 1 mL). The cells were lysed with ice cold radioimmunoprecipitation assay (RIPA) buffer (Thermo Fisher Scientific Inc., 500 µL), and radioactivity content was determined by gamma counter (1282 Compugamma; LKB, window set to channels 35-110 to measure ^{201}Tl gamma emissions). Cell uptake was also performed following [^{201}Tl]TlCl

incubation (100 kBq, 10 µL) for 15 min and 1 h. Cells were harvested and radioactivity measured as described above.

Figure 5 shows a cell uptake experiment where [^{201}Tl]Tl-pypa-PSMA or [^{201}Tl]TlCl was incubated with DU145 PSMA positive or PSMA negative cells. There is no difference between PSMA positive and PSMA negative cells, and uptake can be reduced using excess KCl, suggesting [^{201}Tl]Tl^{+} enters the cell and not the bioconjugate. This is supported by negligible effects of the PSMA inhibitor PMPA on the cell uptake.

NMR spectra of compounds 1-7

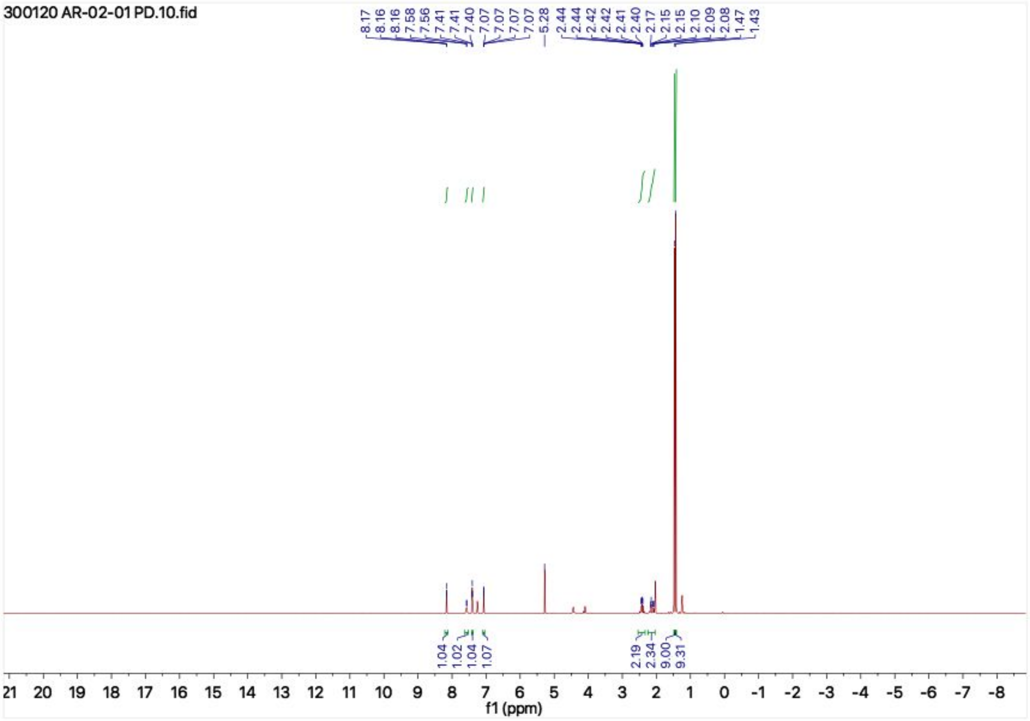

*Figure S9 – Compound **1** ^{1}H NMR spectrum (400 MHz, 298 K, $CDCl_3$)*

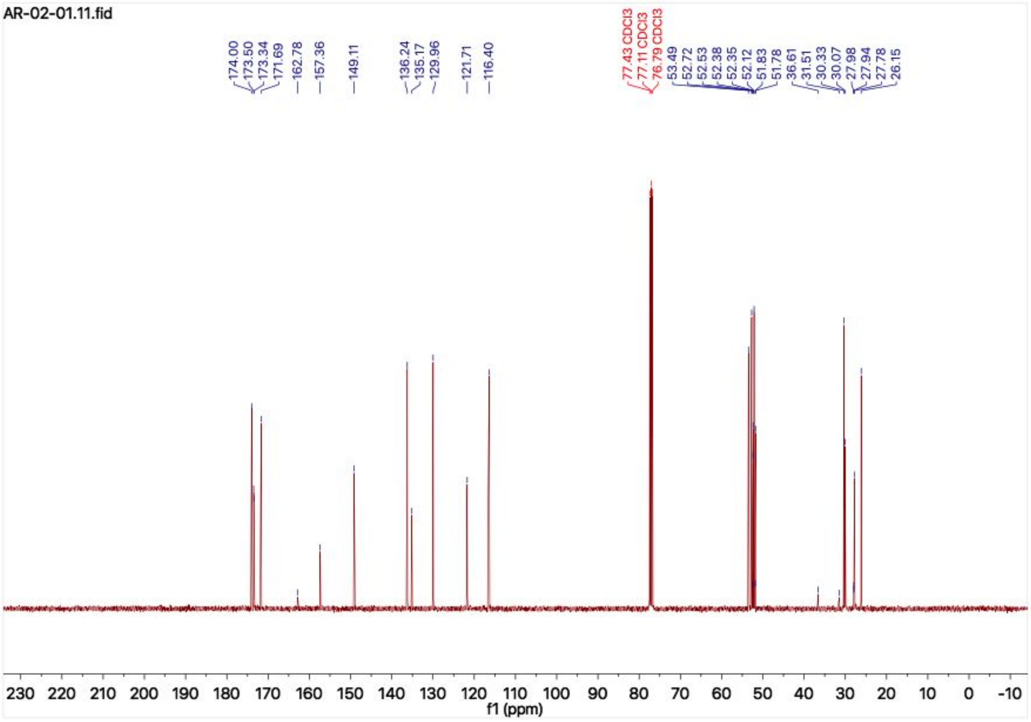

*Figure S10 - Compound **1** ^{13}C NMR spectrum (101 MHz, 298 K, $CDCl_3$)*

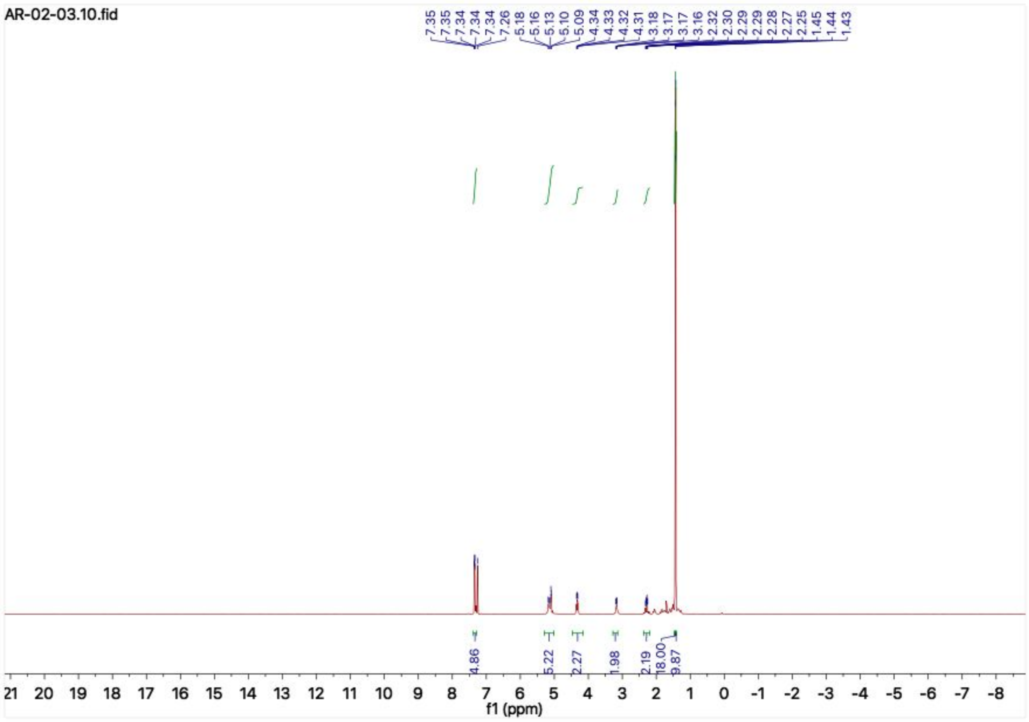

*Figure S11 – Compound **2** ^{1}H NMR spectrum (400 MHz, 298 K, $CDCl_3$)*

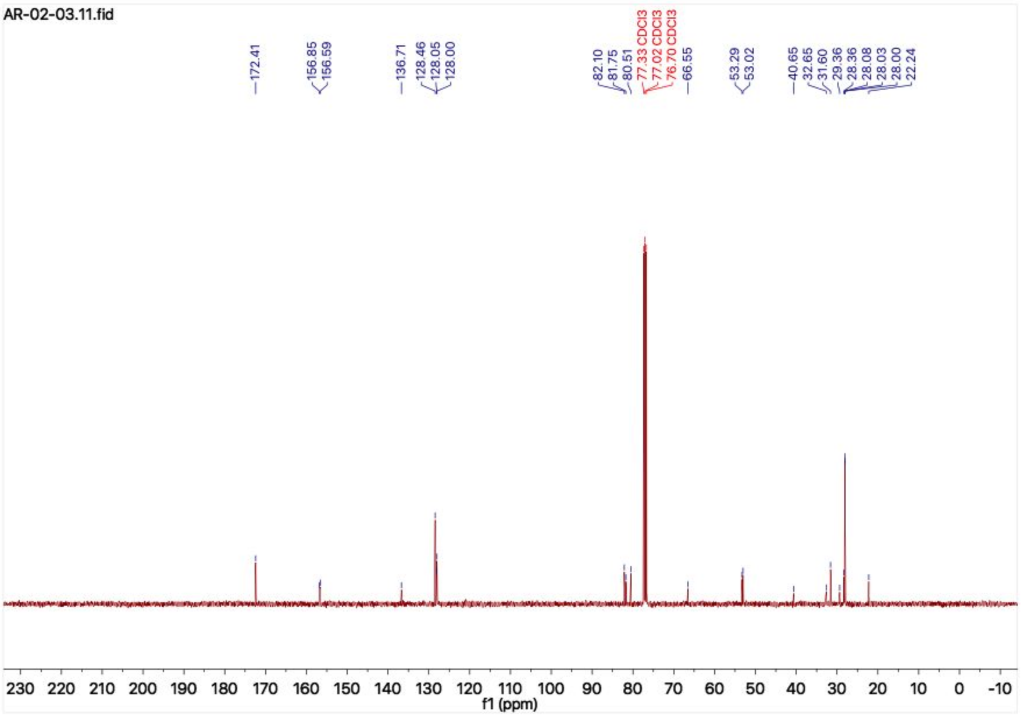

*Figure S12 – Compound **2** ^{13}C NMR spectrum (101 MHz, 298 K, $CDCl_3$)*

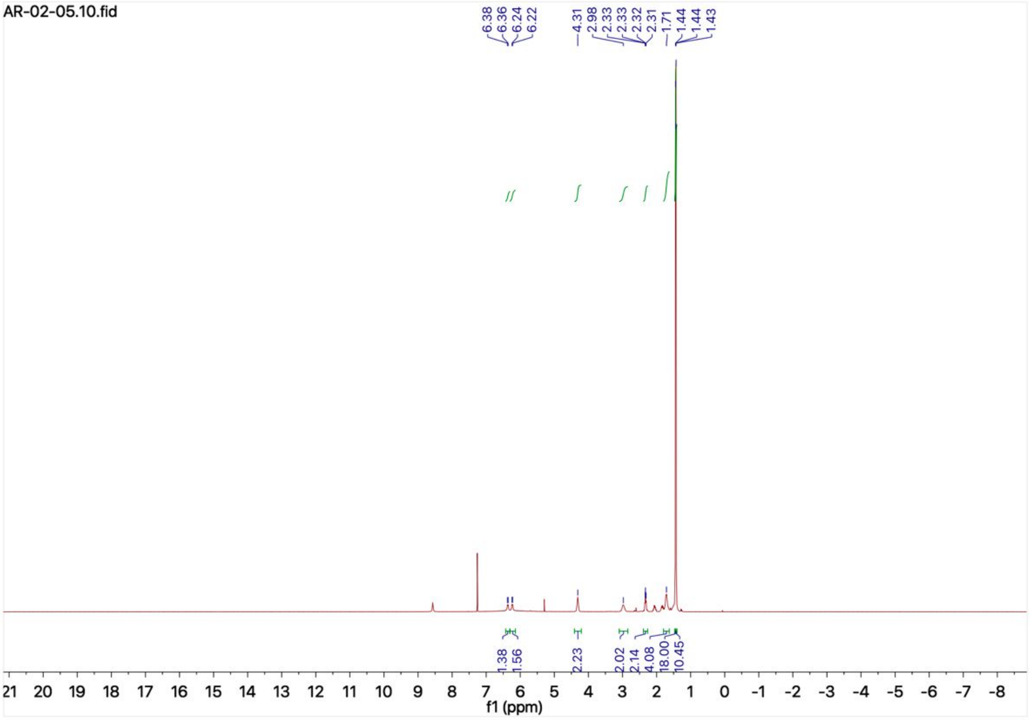

*Figure S13 – Compound **3** ^{1}H NMR spectrum (400 MHz, 298 K, $CDCl_3$)*

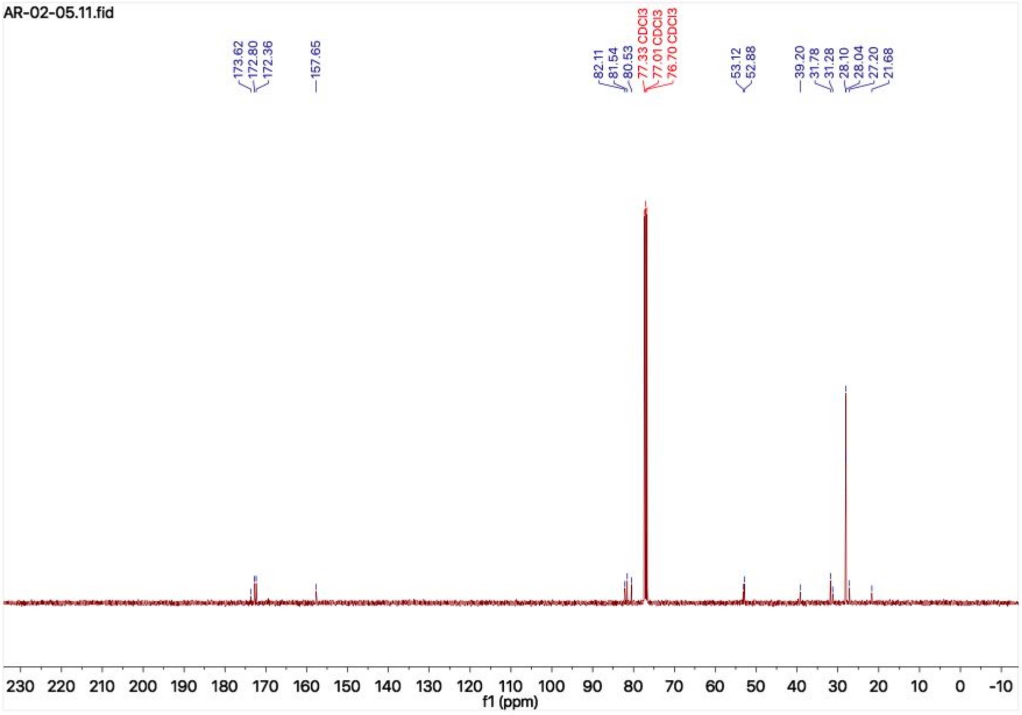

*Figure S14 – Compound **3** ^{13}C NMR spectrum (101 MHz, 298 K, $CDCl_3$)*

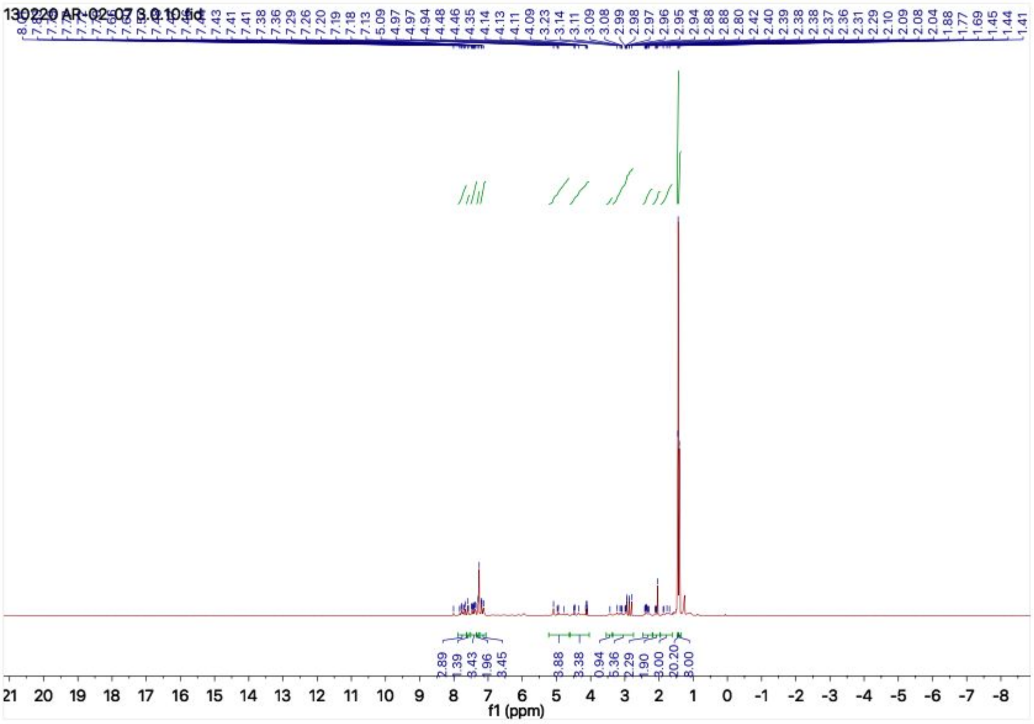

*Figure S15 - Compound **4** ^{1}H NMR spectrum (400 MHz, 298 K, $CDCl_3$)*

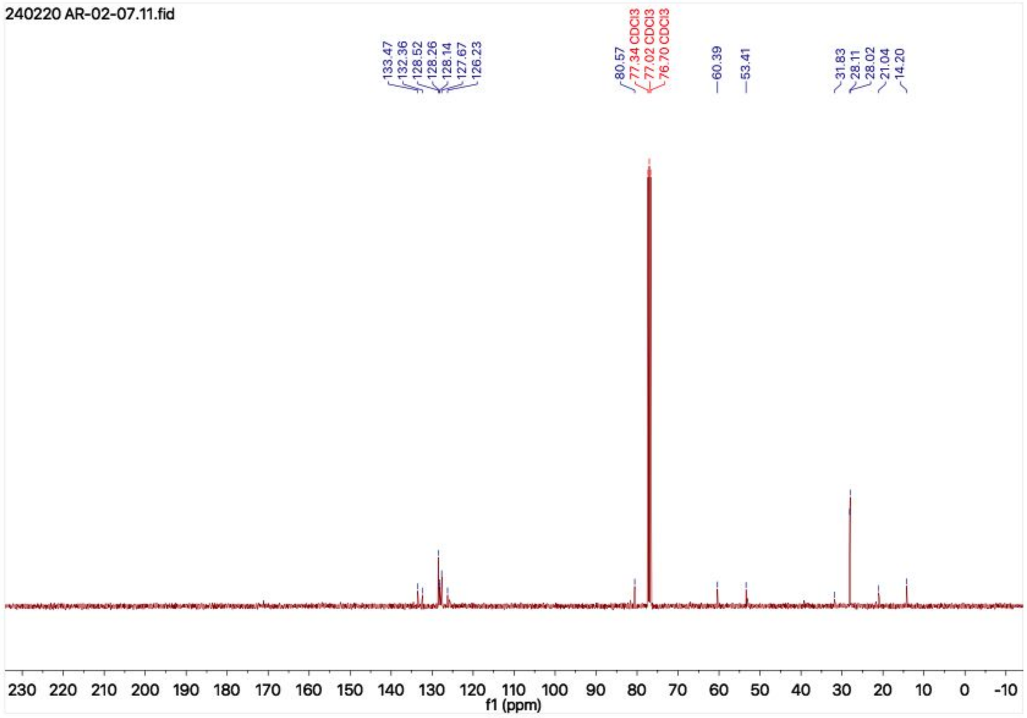

*Figure S16 - Compound **4** ^{13}C NMR spectrum (101 MHz, 298 K, $CDCl_3$)*

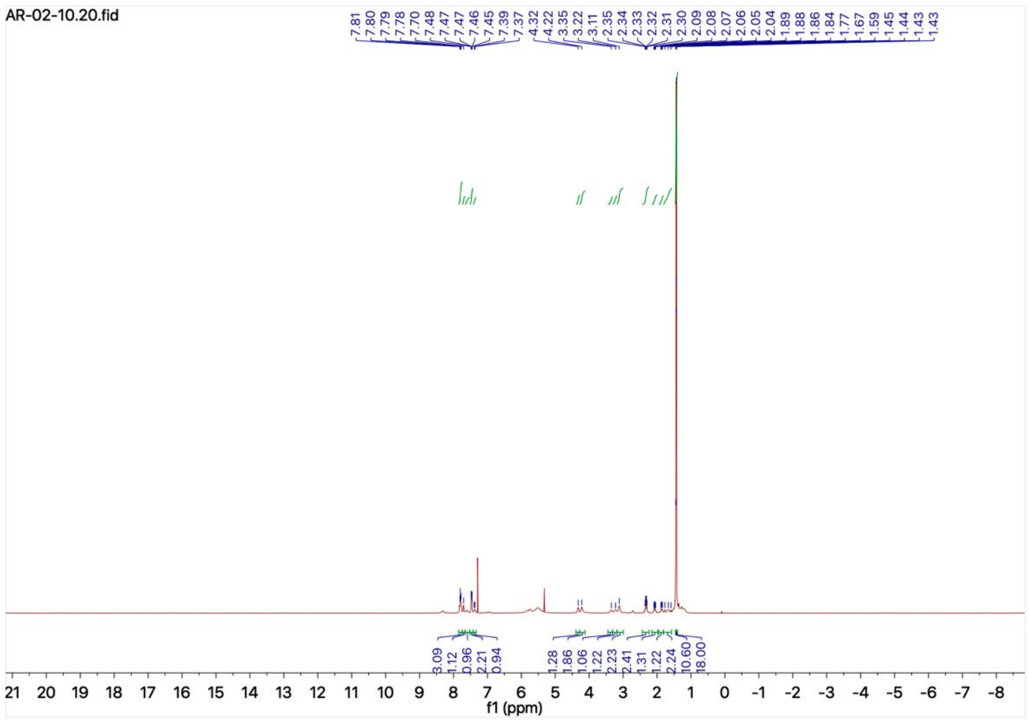

*Figure S17 - Compound **5** ^{1}H NMR spectrum (400 MHz, 298 K, $CDCl_3$)*

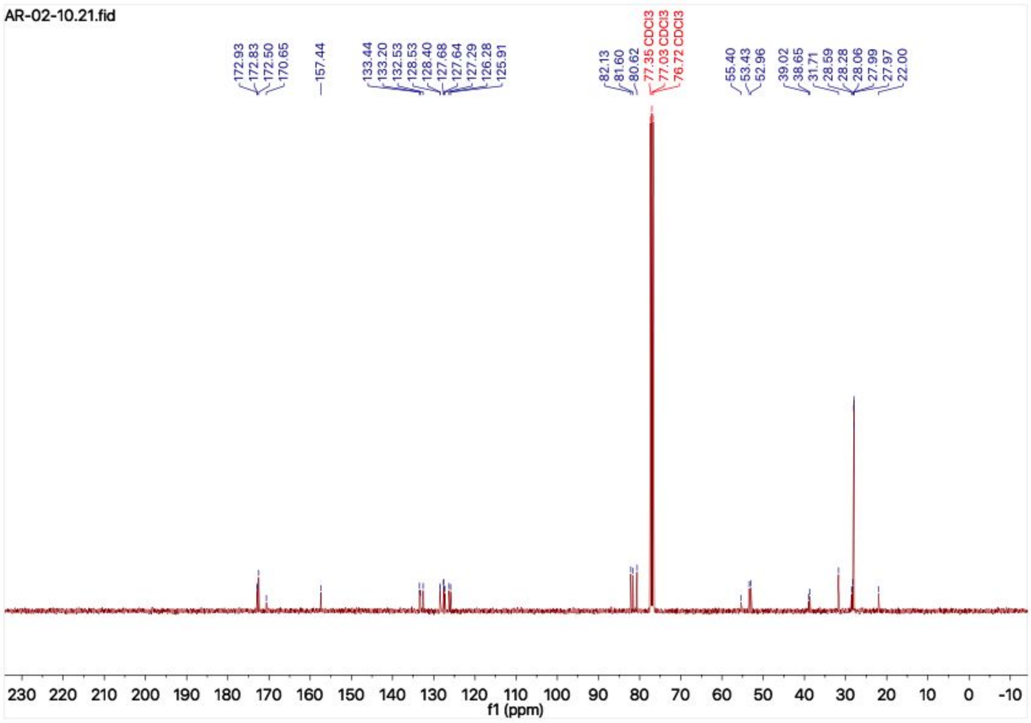

*Figure S18 - Compound **5** ^{13}C NMR spectrum (101 MHz, 298 K, $CDCl_3$)*

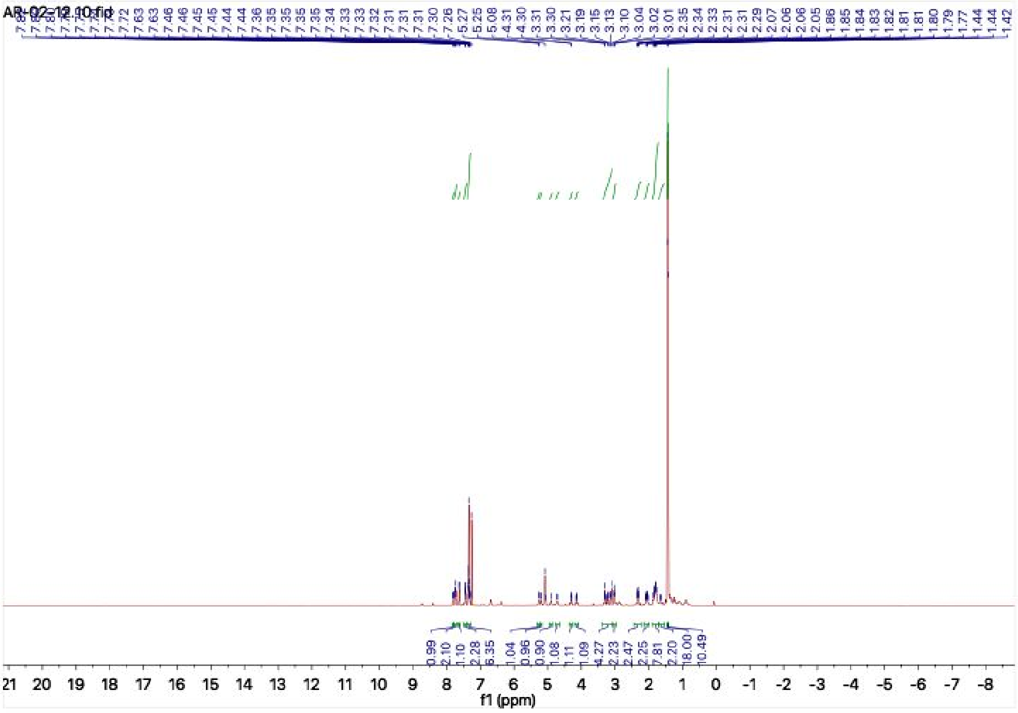

*Figure S19 - Compound **6** ^{1}H NMR spectrum (400 MHz, 298 K, $CDCl_3$)*

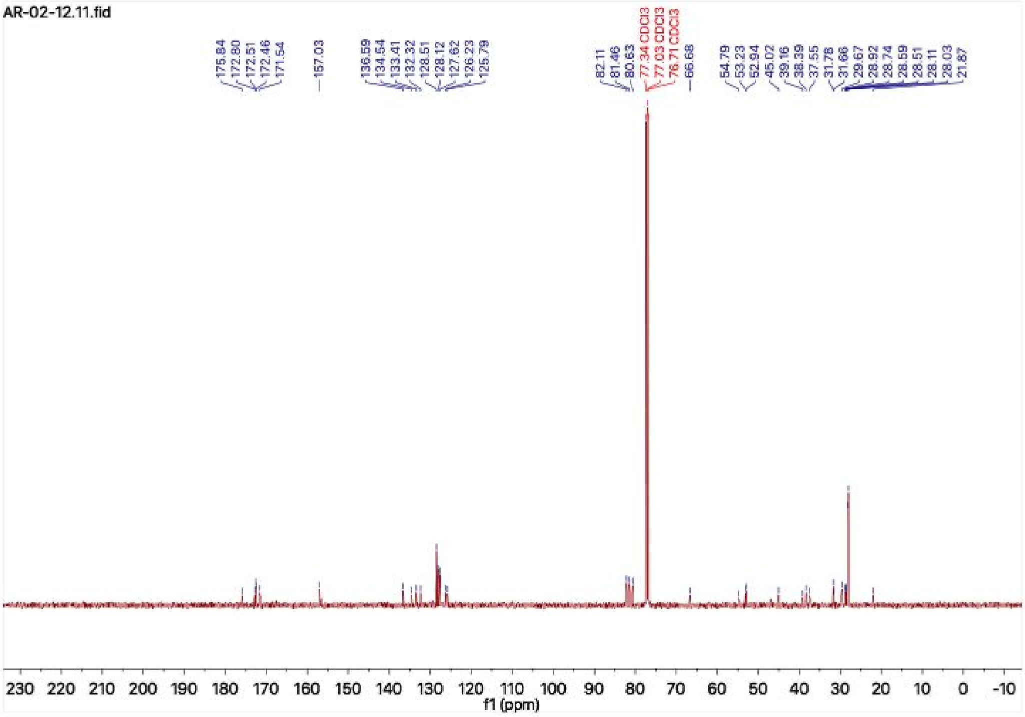

*Figure S20 - Compound **6** ^{13}C NMR spectrum (101 MHz, 298 K, $CDCl_3$)*

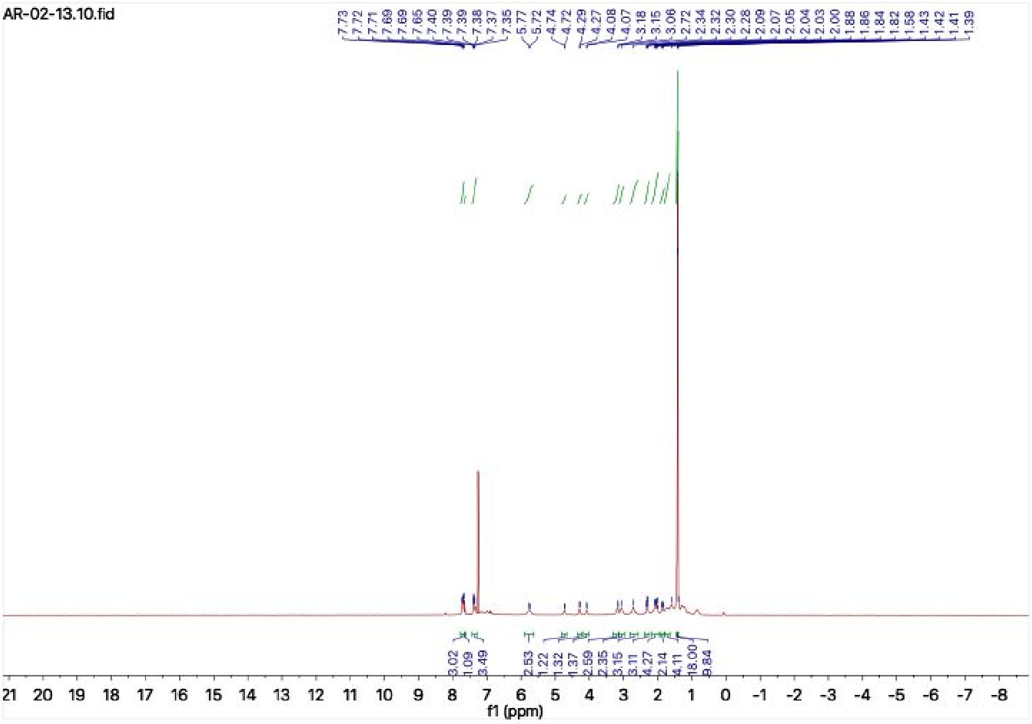

*Figure S21 - Compound **7** ^{1}H NMR spectrum (400 MHz, 298 K, $CDCl_3$)*

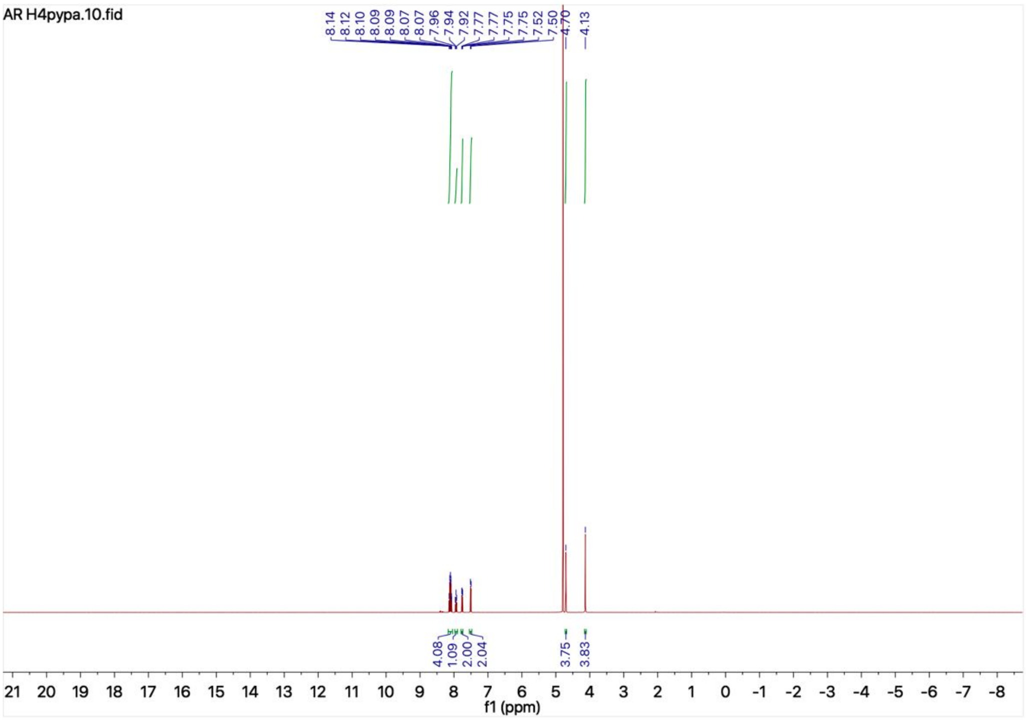

Figure S22 – H_4pypa ^{1}H NMR spectrum (400 MHz, 298 K, $CDCl_3$)

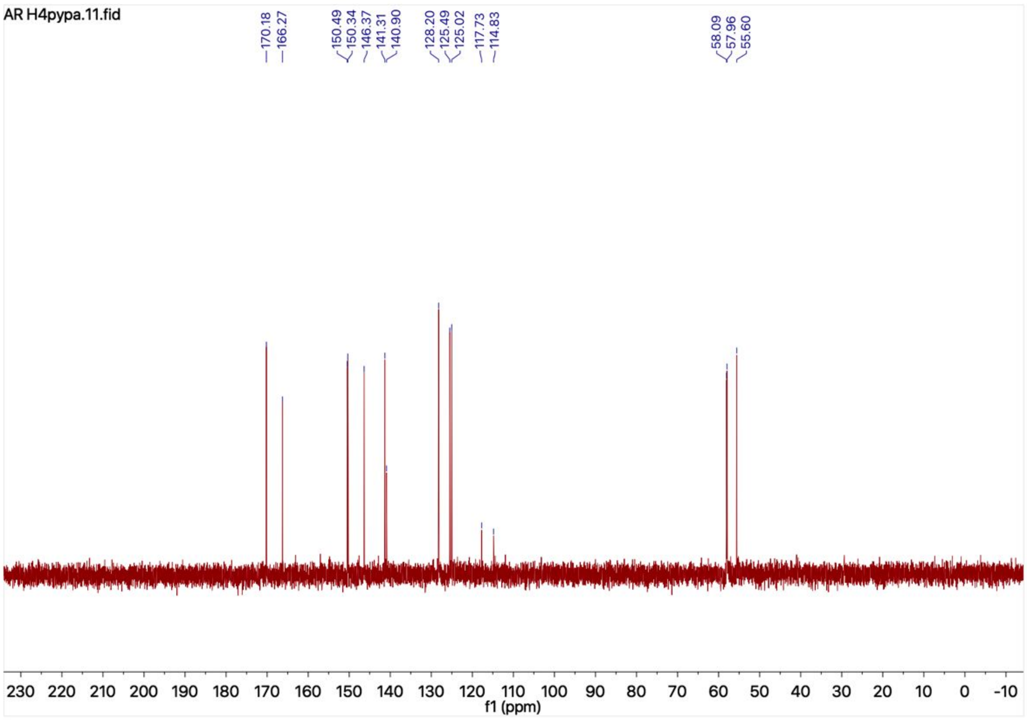

Figure S23 - H_4pypa ^{13}C NMR spectrum (101 MHz, 298 K, $CDCl_3$)

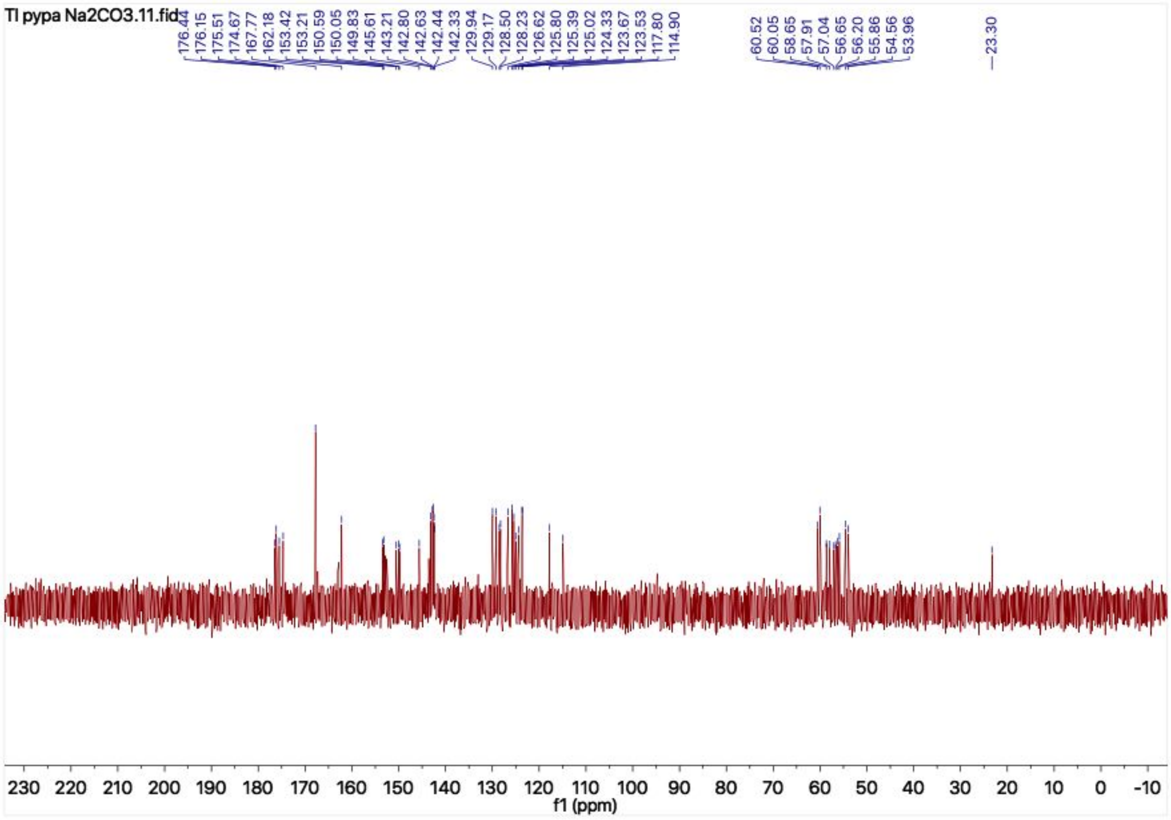

Figure S24 – [^{nat}Tl]Tl(III)-pypa ^{13}C NMR spectrum (101 MHz, 298 K, $CDCl_3$)

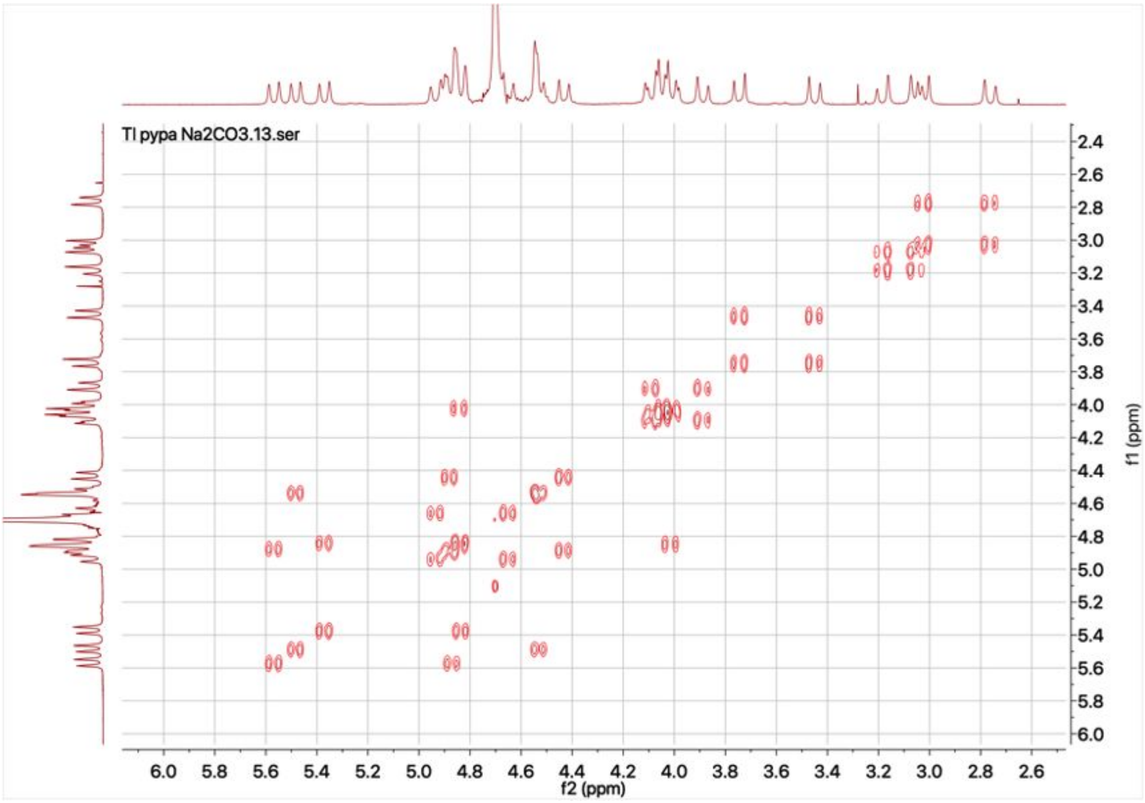

Figure S25 – [natTl]Tl-pypa COSY NMR spectrum

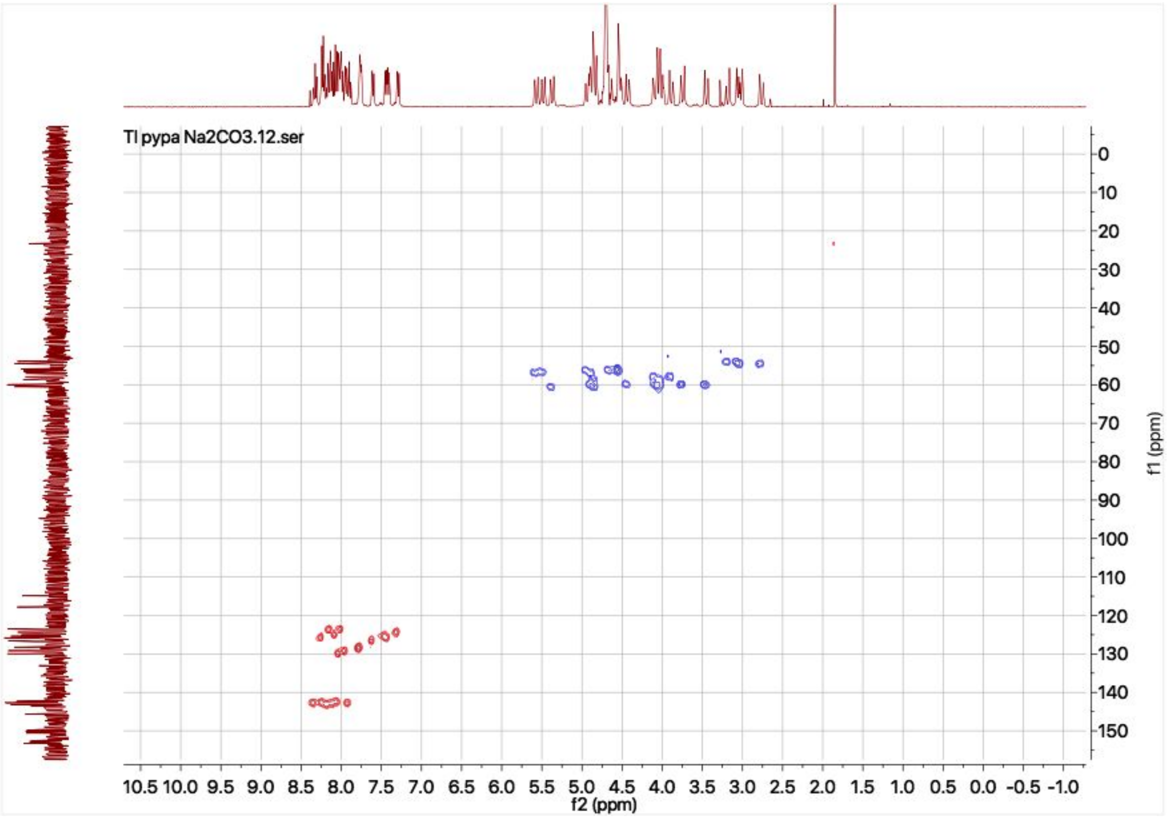

Figure S26 – [natTl]Tl-pypa HSQC NMR spectrum

High resolution mass spectra

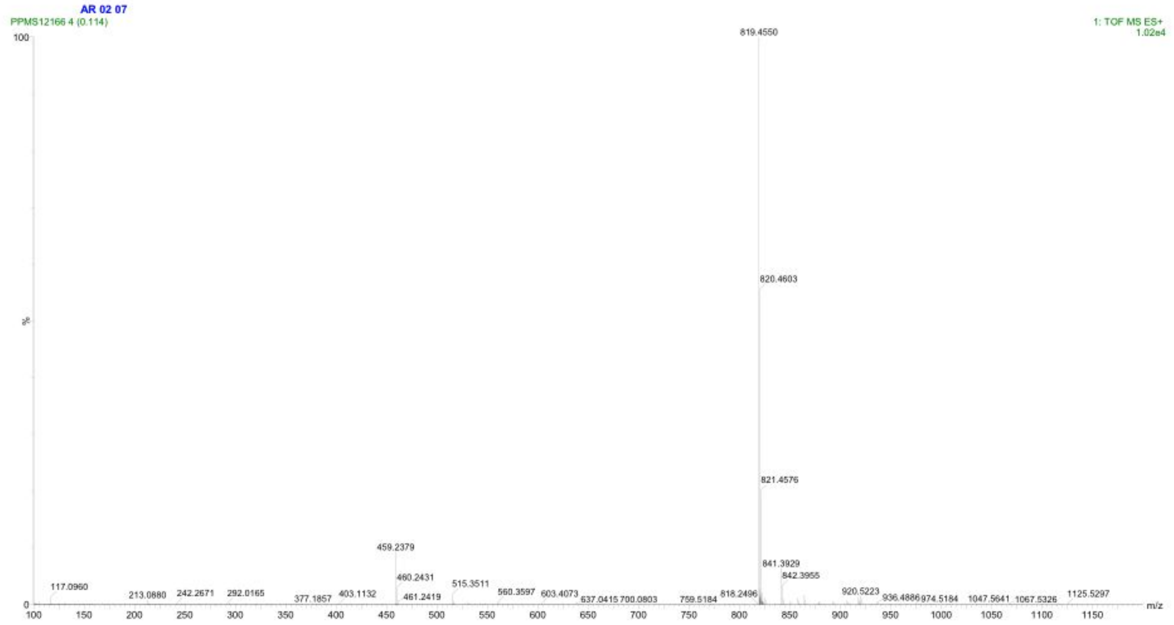

*Figure S27 – Compound **4** high resolution mass spectrum*

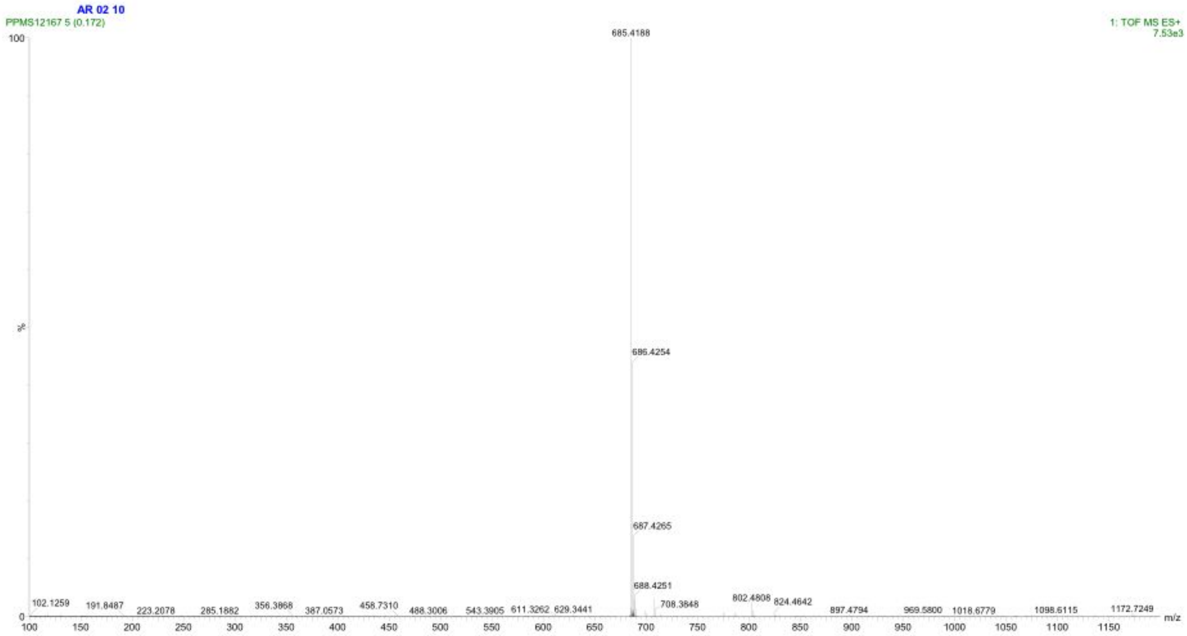

*Figure S28 - Compound **5** high resolution mass spectrum*

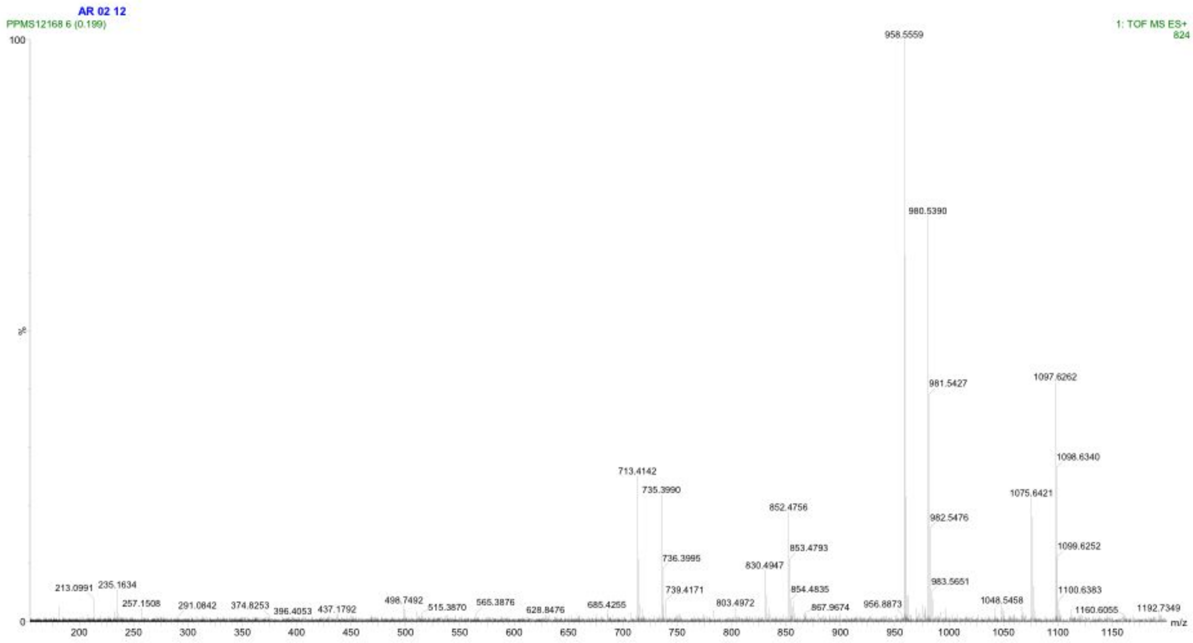

*Figure S29 - Compound **6** high resolution mass spectrum*

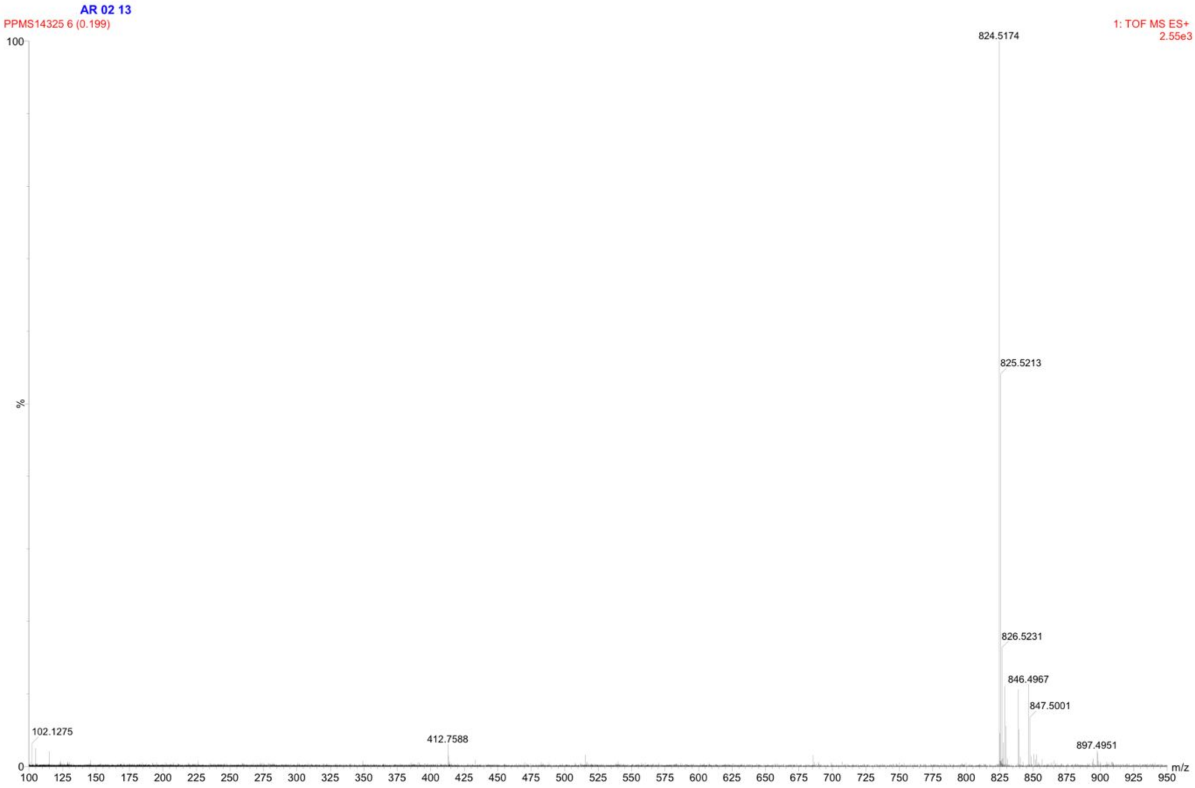

*Figure S30 - Compound **7** high resolution mass spectrum*

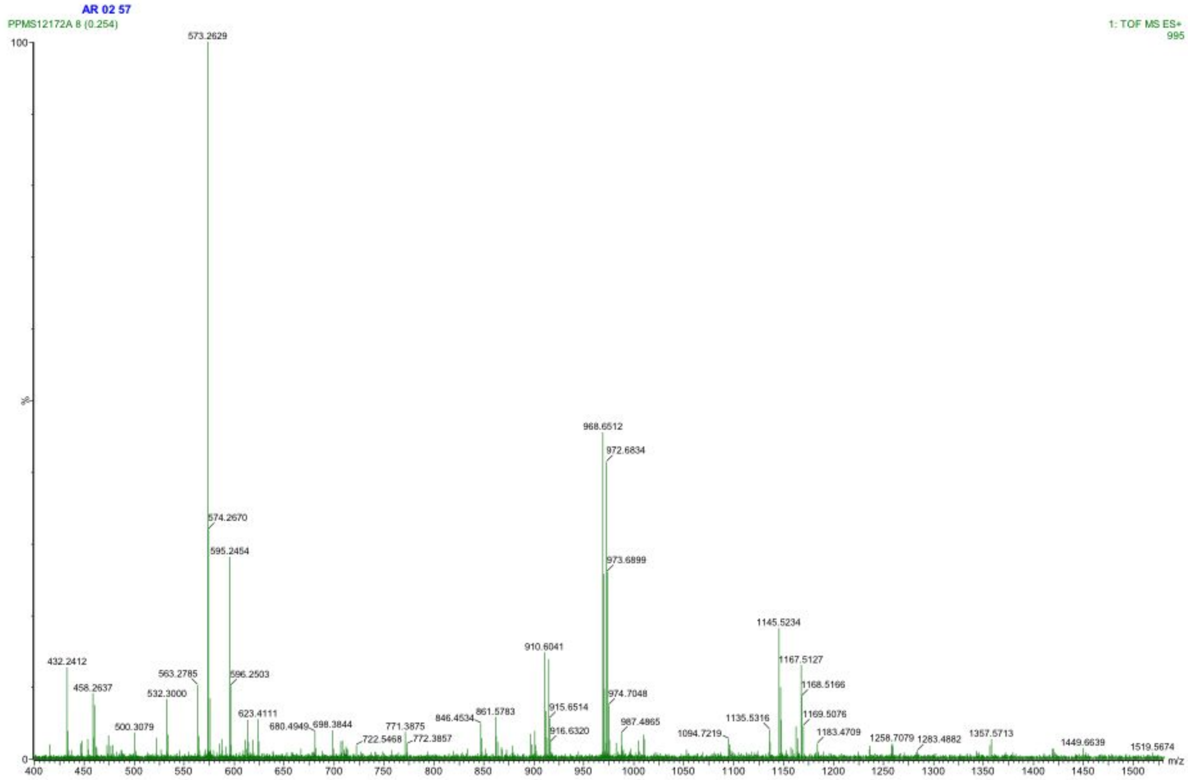

*Figure S31 - Compound **9** high resolution mass spectrum*

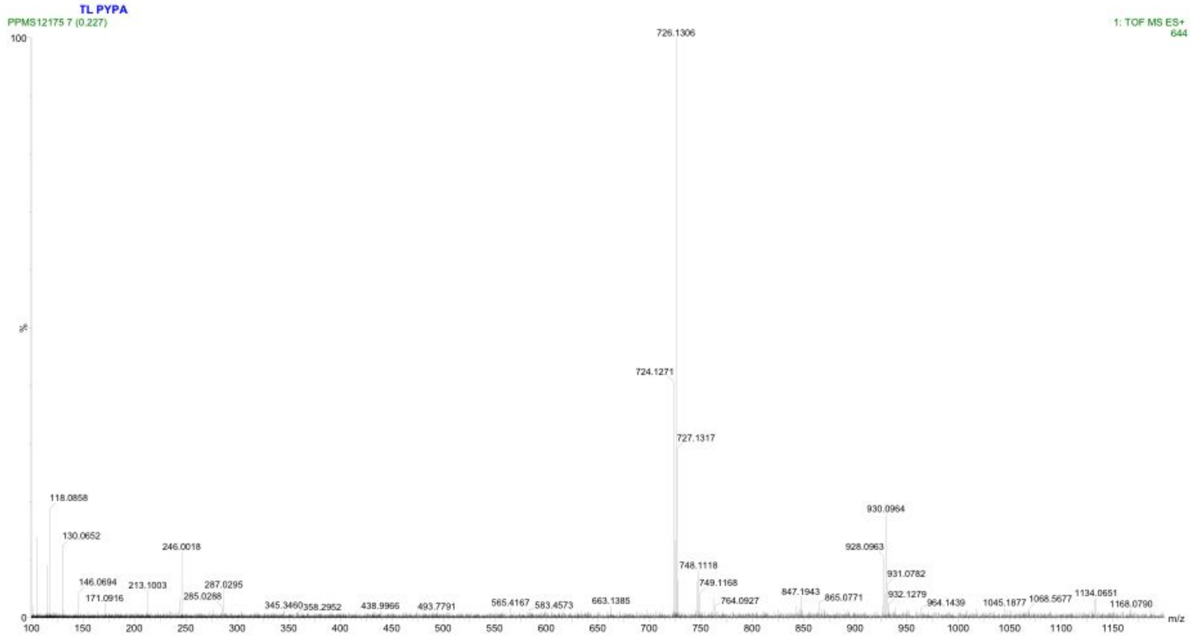

*Figure S32 – **[Tl(pypa)]** high resolution mass spectrum*

X-ray crystallography

The X-ray crystal structure of [Tl(Hpypa)][TlCl$_3$(H$_2$O)]. The unit cell was found to contain two Tl(III) centres: one sitting at the centre of the pypa ligand while the other is a $TlCl_3$ unit (presumably from the starting material) located at the extremity and coordinated by one carboxylate group from the pypa ligand and another H_2O molecule. Crystals were grown directly from a reaction mixture of $TlCl_3$ and pypa ligand at pH 2. One chloride ligand was found to be disordered and was modelled over two positions (Cl3A and Cl3B) with relative occupancy of 86% and 14%. These were modelled anisotropically and isotropically, respectively.

$C_{25}H_{24}Cl_3N_5O_9Tl_2$, M = 1053.58, triclinic, P-1 (no. 2), a = 9.0552(4), b = 11.4821(7), c = 15.7252(10) Å, α = 71.114(6), β = 75.497(5), γ = 83.697(4)°, V = 1496.94(16) Å^3, Z = 2, D_c = 2.337 g cm^{-3}, μ(Cu-Kα) = 23.432 mm^{-1}, T = 173 K, colourless plates, 5668 independent measured reflections (R_{int} = 0.0365), F^2 refinement, R_1(obs) = 0.0372, wR_2(all) = 0.0985, 4629 independent observed absorption-corrected reflections [$|F_0| > 4\sigma(|F_0|)$, $2\theta_{max}$ = 147°], 404 parameters. CCDC 2115641.

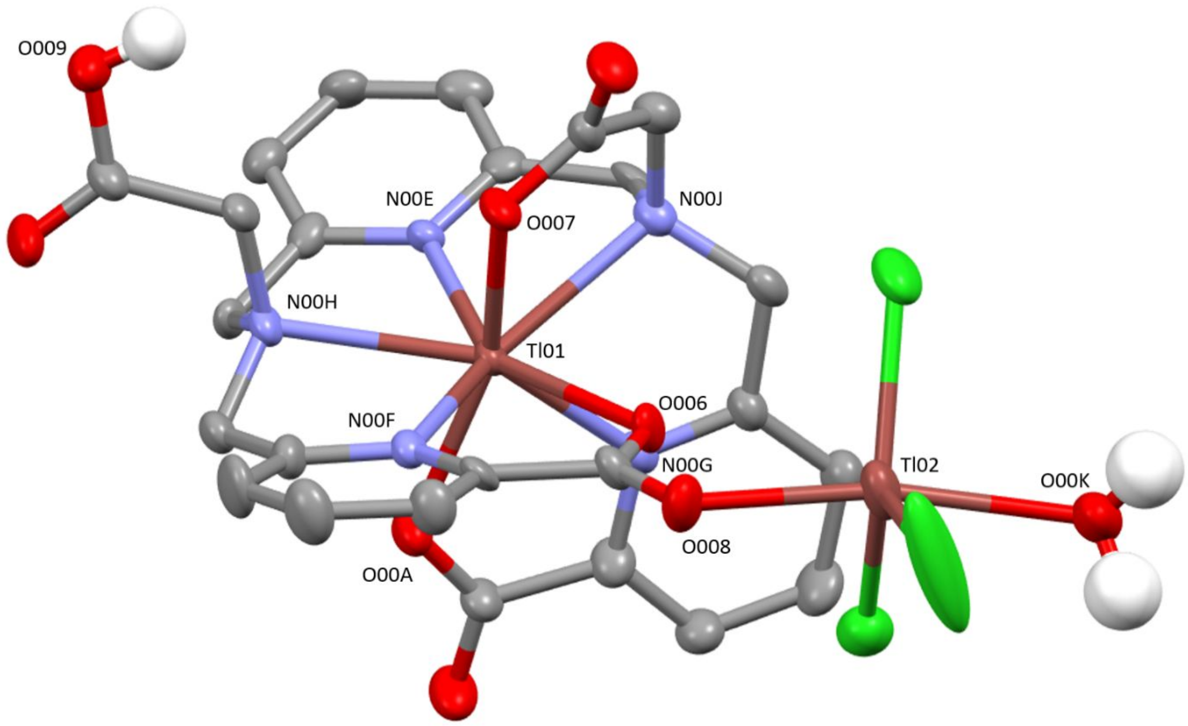

Figure S33 - Crystal structure of [Tl(Hpypa)][TlCl$_3$(H$_2$O)]. Selected hydrogens removed for clarity. Thermal ellipsoids drawn at 50% probability.
